# An automatic classification of breast cancer using fuzzy scoring based ResNet CNN model

**DOI:** 10.1038/s41598-025-07013-6

**Published:** 2025-07-01

**Authors:** Nisha Y., Jagadeesh Gopal

**Affiliations:** School of Computer Science Engineering and Information Systems, Vellore Institute of Technology, Vellore, India

**Keywords:** Breast cancer, Feature extraction, Statistical correlation analysis, Fast discrete wavelet transform, Adaptive grey wolf optimization algorithm, Hybrid deep learning, Fuzzy scoring ResNet, Cancer, Diseases, Health care

## Abstract

The expansion rate of medical data during the past ten years has rapidly expanded due to the vast fields. The automated disease diagnosis system is proposed using a deep learning (DL) algorithm, which automates and helps speed up the process efficiently. Further, this research concentrates on improving computation time based on the detection process. So, this research introduces a hybrid DL model for improving prediction performance andreducing time consumption compared to the machine learning (ML)model.Describing a pre-processing method utilizing statistical co-relational evaluation to improve the classifier’s accuracy.The features are then extracted from the Region of Interest (ROI) images using the wrapping technique and a fast discrete wavelet transform (FDWT). The extracted curvelet coefficients and the turn-time difficulty are too excessive to be categorized. Utilizing swarm intelligence, the Adaptive Grey Wolf Optimization Algorithm (AGWOA) was presented to reduce the time difficulty and choose the key characteristics. Here, it introduces a new building block identified as the Fuzzy Scoring Resnet-Convolutional Neural Network(FS-Resnet CNN) framework to optimize the network. The performance of the proposed model was assessedutilizing metrics such as recall, precision, F-measure, and accuracy.Furthermore, the suggested framework is computationally effective, less noise-sensitive, and efficiently saves memory. The simulation findings indicate that the suggested framework has a higher detection rate than the existing prediction model.

## Introduction

With the greatest rates of death and disability among women, BC is among the worst affected by malignancies. However, BC may be treated in almost 90% of cases when detected early. Women’s lives can, therefore, be saved by early identification and treatment of BC^1^. Whether the disease is malignant (invading and life-threatening) or benign (localized and non-invading), an accurate diagnosis can assist a patient in eliminating their chance of developing BC. Various techniques, such as mammography, CT scans, and histopathological image evaluation (biopsy images), are frequently employed for diagnosis. Histopathological imaging may diagnose BC with excellent mammography results^2^. Histology image evaluation systems are typically composed of hardware and software, and they might be further subdivided into two sequential components: (1) image generation and tissue setup, and (2) image processing evaluation. Awareness about BC and appropriate screening or diagnosis are two crucial factors in lowering the fatality rate amongst women.

Most medical imaging techniques for diagnosing BC include medical screens, MRI, and molybdenum target X-ray imaging^3^. Molybdenum mammography is a medical screening that offers several benefits, including low cost, ease of use, and minimal risk to patients. In addition to helping radiologists diagnose breast cancer more quickly, automatic computer-aided mammography screening of BC can optimize the precision of BC detection and reduce expenses. By evaluating the efficacy of prospective variables that were identified employing mammography image extraction^4^ in medical image processing, the ML methods forcategorizing benign or malignant breast masses have generated debate. The visual characteristics of tissue removed from a patient are utilized to identify BC. Next, step-by-step evaluations include collecting the specimen, tumor cell nuclei, image pre-processing, image segmentation, and feature extraction determined by size, texture, and categorization.

The method of precisely expressing vast data with fewer resources is known as feature extraction. The main issue in analyzing complex data is the number of factors included^5^. Large-scale variable evaluation typically calls for high memory and processing resource requirements or a classification technique that overfits the training set and performs badly on fresh samples. A common abbreviation for techniques to create variation combinations to circumvent these issues and still accurately describe the data is feature extraction^6^. A surface’s tactile or visual texture. The goal of texture evaluation is to discover a distinctive method for encapsulating the fundamental properties of patterns in a more straightforward yet distinctive form, which can then be applied to reliable and precise object categorization and segmentation. Few systems incorporate onboard textural feature extraction, even though texturing is crucial for assessing images and identifying patterns^7^. A gray-level co-occurrence matrix was developed in this research to acquire statistical texture features. It is possible to extract several texture features from the GLCM. Four second-order properties are calculated: entropy, inverse difference moment, correlation, and angular second moment. These four measurements provide the high discrimination accuracy needed for motion picture estimates.

Many academics are interested in DL, a rapidly developing technology in ML.CNN has extensively accomplished large-scale image and video recognition. Images of BC pathology are classified utilizing AlexNet for both benign and malignant classifications^8^. Their categorization scores are 6% better than conventional ML classification methods. DeCAF features are retrieved utilizing the previously learned CNN reuse as a feature vector. The classifier trained for the latest classification task will receive the DeCAF feature as input. A CNN multiple instance learning (MIL) system was suggested. A new pooling layer was created, facilitating the aggregation of the most informative characteristics from the patches that made up the entire slide, exclusive of requiring global slide coverage^9^. Currently, automatic CNN-based classification of abnormal BC images remains a challenging issue. The following are the particular causes: (1) The quantity of CNN variables rises quickly due tothe system’s ongoing deepening, which makes the algorithm susceptible to over-fitting. CNN training often requires many histological pictures of BC as training data to minimize the over-fitting danger. But getting many annotated BC photos is pricey^6^. Consequently, it is necessary to lower the danger of overfitting in the algorithm when there is a shortage of BC imaging data by utilizing data augmentation techniques and lowering CNN settings. (2) It is commonly recognized that several hyperparameters substantially affect the CNN algorithm’s efficiency. It is frequently essential to manually modify the learning rate variables throughout the training of models to achieve higher accuracy^10^, making it difficult for non-expert users to employ the technique in practical situations. A lightweight CNN component was created utilizing the features of BC histological images to lower the CNN training settings. This network was then created to classify BC histopathology images.A new pipeline for feature extraction, classification techniques, and BC cell identification was added to address the aforementioned issues and improve the precision of cancer image categorization.So, this research introduces a hybrid DL model for improving prediction performance andreducing time consumption compared to the MLmodel.Here, the MATLAB model is used to detect breast cancer shows the Comparison of Existing Breast Cancer Classification Methods Table 1 shows the Comparison of Existing Breast Cancer Classification Methods.

**Table 1:** Comparison of existing breast cancer classification methods.

Technique	Dataset used	Accuracy (%)	Feature extraction	Optimization/enhancement	Novelty
SVM + PCA	WBCD	94.2	PCA-based reduction	None	Traditional ML pipeline
CNN	BreakHis	96.1	End-to-end CNN	None	Deep learning approach
Hybrid CNN + GWO	MIAS	97.3	CNN + handcrafted features	GWO	Bio-inspired optimizer
ResNet + GA	WBCD + custom	98	Deep ResNet	Genetic Algorithm	Genetic tuning improves generalization
FS-ResNetCNN + AGWOA	BreakHis + WBCD	98.7	Fuzzy scoring + ResNet	Adaptive GWO	Hybrid fuzzy-deep model with adaptive metaheuristic

The main contribution of the paper is.

- Introducing a novel Fuzzy Scoring ResNet-CNN (FS-ResNet CNN) framework that combines fuzzy logic with deep residual networks to enhance the precision of breast cancer image classification.
- Utilizing Fast Discrete Wavelet Transform (FDWT) for effective multi-level feature extraction and Adaptive Grey Wolf Optimization Algorithm (AGWOA) for dynamic hyperparameter tuning and performance optimization.
- This study demonstrates improved classification accuracy, noise resilience, and faster convergence compared to traditional CNN-based models through comprehensive experimental validation.
- Ablation Studies show the performance impact of each module (e.g., fuzzy scoring vs. no. fuzzy scoring, with vs. without AGWOA).
- The complexity vs. Performance Trade-off quantifies the gains in accuracy or speed relative. To model complexity and training time.
- Comparative Baselines benchmark against similar hybrid models (e.g., CNN + PSO, ResNet + GA) to validate the proposed method’s superiority.

The residual section is arranged as follows:Section"Related work"analyzes recent breast cancer detection methods. Section"Proposed methodology"defines the suggested method. Section"Attacking prey (exploitation):"explains the test outcomes of the suggested algorithms. Section"Search for prey (exploration):"deals with the conclusion and future work.

## Related work

The identification of BC is the primary goal of research on CAD concerning BC. This section addresses the aids and limitations of the recent methods for detecting BC.

A CAD technique was presented in^10^, and the system includes image enhancement, structural distortion identification, image retrieval, mass identification, bilateral asymmetry finding, calcification finding, and structural distortion identification. In^11^, a computerized approach for sensing breast masses utilizing the n-technique is recommended, in which the DFO swarm optimization process is employed to appropriately create the initial seed points and thresholds. Utilizing GLCM and GLRLM approaches, the texture characteristics are retrieved from the segmented images and placed into an FFNN classifier trained using a backpropagation method. Utilizing pictures taken from the DDSM database, the efficacy of the recommended detection is assessed. The ROC technique is employed to evaluate the outcomes of the suggested pixel-based strategy compared to those of other region-growing techniques. The suggested system’s sensitivity and specificity were attained at 98.1% and 97.8%.

A mass detection technique was created using CNN and unsupervised ELM clustering^12^. Combine deep, morphological, texture, and density data to create feature sets. To differentiate between malignant and benign, an ELM classifier is created. The suggested approach for mass detection and BC categorization is proven accurate and effective through extensive testing. A non-parametric method for utilizing mammography to detect, diagnose, and cure BC was introduced in^13^. The detection issue exploits the shared data betweenthe picture pixel intensity and the intended areas. It suggests a non-parametric density estimate that employs curve evolution techniques to define the theoretical data optimization issue and derive the correlated gradient flows. The application of level-set procedures obtains the ensuing evolution. Twelve groups of mammography images, 156 sets total, having both malignant and/or normal images, were subjected to the method. The outcomes demonstrated that the optional method had an accuracy of 94.937% in detecting BC.

In^14^, an approachable and understandable method was suggested. The suggested system mines contrast features among normal and cancerous pictures in a weak-supervised way with minimal annotation data and produces a probabilistic map of abnormalities to validate its assumptions. A fully convolutional autoencoder learns the dominating structural trends among normal picture patches. One-layer neural networks and one-class SVM are employed to find and assess patches that do not match the features. Utilize a public image set of BC to test the suggested approach. According to the findings discussed with a senior pathologist, the suggested approach works better than the current one. The probability map that was generated has the potential to enhance pathology practice by offering a visual representation of verification data and facilitating a deeper comprehension of data-driven diagnosis approaches. A frame mammography tumor identification system with the optimal hybrid classifier was presented in^15^. The functional stages of the suggested BC diagnosis method include image pre-processing, tumor classification, feature extraction, and identification. By employing a median filter, the input mammogram’s noise is removed. Additionally, an FC-CSO is required for the optimum region growth segmentation, which separates the tumor from the image. Feature extraction, which attempts to extract features like GRLM and GLCM, is performed after tumor segmentation. The CNN and RNN are two DL systems. While GLRM and GLCM are accepted as input to RNN, the tumor split binary image allows the input to the CNN. The research outcome demonstrates that the AND operation of two classifier outputs will produce diagnostic accuracy superior to that of traditional algorithms. In^16^, a system for organizing mammography images into benign, normal, and malignant categories was created through a CNN to identify BC. By supporting specialists in the detection and categorization of BC, BCDCNN attempts to expedite the diagnosis procedure. Pre-processing is done on a sequence of mammography pictures to change appropriate CNN classifier parameters and transform an individual’s visual image into a computer visual image. All modified photos are then used as training data by the CNN classifier. After that, a simulation to identify the mammography picture will be developed with the CNN classifier. BCDCNN has increased the classification accuracy of mammography pictures when compared to MCCNN. The outcomes demonstrate that the suggested approach is more accurate than other techniques. The accuracy of the mass only and all arguments has increased from 0.75 to 0.8585 and from 0.608974 to 0.8271.

In^17^, a DL method was suggested to identify BC in DCE-MRI images automatically. A complete CNN structure, utilizing U-net. Every single breast slice can be handled by the trained model for both identification and segmentation. The dataset of 86 DCE-MRI slices, obtained from both pre-and post-chemotherapy, from 43 patients with local BC was utilized in this investigation. A skilled radiologist has manually annotated the data. With a mean IoU of 76 14%, the algorithm was trained and verified on 85% and 15% of the data, respectively. In^18^, a 3D-DCE-MRI was gathered for 42 patients suffering from BC. Fourteen of these individuals reacted favorably to chemotherapy, whereas the remaining twenty-eight did not, per the pCR ground truth. Patients were categorized as responsive or non-responsive utilizing a CNN algorithm. Two CNN branches were utilized in this design to create this category. In three perspectives, the inputs to this creation are two aligned DCE-MRI cropped volumes obtained before and after the first chemotherapy. The data was divided into training (80%) and validation (20%). A cross-validation technique was employed to assess the suggested CNN framework. The accuracy and AUC of the framework were utilized to evaluate its efficacy. In^19^, a DL classifier, in which algorithms are constructed from scratch andutilize transfer learning,is further subcategorized by deep classifiers. The efficacy of various categorization systems was evaluated with various parameters, and some suggestions are presented for future studies that should come before this research. In^20^, it utilized radiomics to thoroughly evaluate the radiological pictures to build an automated classification system and determined 70 radiomic features, such as the lesion’s shape, spicula, and texture data, by extracting an evaluation region concentrated on the lesion. Four classifiers were trained to employ the gathered radiomic characteristics, and the resulting accuracy was evaluated. An accurate classifier was employed to get the final classification outcome as an output. The efficacy of the suggested approach was evaluated utilizing 24 instances with biopsy-confirmed pathological diagnoses, and the categorization outcomes were evaluated according to whether LASSO was used for dimension reduction. Consequently, the outcomes were obtained when the SVM was utilized as a classifier, with a correct detection rate of 84% for malignant and 55% for benign tumors. These findings suggest that the suggested approach might assist in more precisely detecting conditions that are challenging to categorize just on pictures.

Jing Zhang et al.^28^ suggested the radiomics model with minimal stable, interpretable features for fully automatically classifying breast lesions on multi-parameter MRI. Automated lesion ROI segmentation using an nnUNet-based lesion segmentation model allowed for extracting radiomics characteristics for use in DWI, T2WI, and DCE pharmacokinetic parametric maps. The author used logistic regression and support vector machines to build models based on sequence combinations. Additionally, BI-RADS scores and the efficiency of these sequence combinations were evaluated. The segmentation and classification outcomes were evaluated using the Dice coefficient and AUC. A decision curve analysis (DCA) was used to determine the clinical value.

TARIK AHMED RASHID et al.^29^ proposed the Human epidermal growth factor receptor 2 (HER2) Classification in Breast Cancer using Metaheuristic Optimal Feature Selection With a DL Framework. Reducing model complexity and computational costs while avoiding overfitting is achieved efficiently by the recommended approach. First, the suggested cascaded design converts WSIs to tiled images and uses fast local Laplacian filtering (FLIPF) to enhance contrast. Second, a ResNet50 CNN technique extracts features based on transfer learning. Third, an optimizer from a non-dominated sorting genetic algorithm (NSGA-II) helps select the most informative features. Lastly, a support vector machine (SVM) is used to classify HER2 scores. The HER2GAN and HER2SC datasets demonstrate that the proposed model outperforms current techniques. On the HER2SC dataset, the model obtained 94.4% accuracy, 93.71% precision, 98.07% specificity, 93.83% sensitivity, and a 93.71% F1score.

Mario Verdicchio et al.^30^ recommended a pathologic approach for classifying tumor-infiltrating lymphocytes on breast cancer digital pathology images. The data utilized in this study came from 195 TNBC and HER2 + BC hematoxylin and eosin (H&E)-stained WSIs, and it included 1037 ROIs annotated with tissue compartments and TILs. Following the watershed-based segmentation of tumor-associated stroma into nuclei, 71 pathomic characteristics were recovered from each nucleus and further reduced using Spearman’s correlation filter, a non-parametric Wilcoxon rank-sum test, and the least absolute shrinkage and selection operator. Using five multivariate ML classification models trained using fivefold cross-validation, every candidate nucleus was categorized as TILs or non-TILs based on the relevant attributes. The models were trained in three different ways: (1) without resampling, (2) using the synthetic minority over-sampling approach, and (3) with downsampling. The ROC curves were used to evaluate the models’ prediction performance.

Jacinta Potsangbama and Salam Shuleenda Devia^31^ discussed Transfer Learning with DenseNet121 for classifying Breast Cancer Histopathological Images. A publicly available standard database, the Breast Cancer Histopathological Database (BreakHis), is used to verify the proposed framework design. In the pre-processing phase, data augmentation methods are used. This work uses the DenseNet 121 pre-trained model to extract features and then adds fully connected layers (FCL) to make it even better. The trial shows that the 100X achieves the maximum accuracy of 96.09%. The testing findings demonstrated that, compared to previous efforts, the accuracy improved across the board for all magnification factors.

Abeer Saber et al.^32^ deliberated the optimized ensemble model based on meta-heuristic algorithms to detect and classify breast tumors. The suggested approach uses ensemble pretrained models for data categorization, including support vector machines, feature extractor networks like EfficientNet-B5, dense convolutional network (DenseNet)−121, and more. The chosen pre-trained CNN hyperparameters were fine-tuned to enhance performance using a modified meta-heuristic optimizer. Overall, the EfficientNet-B5 model achieved 99.9% accuracy, 99.8% specificity, 99.1% precision, and 1.0 area under the ROC curve (AUC) when tested on the INbreast dataset, proving its efficacy for BC classification.

Musa Yusuf et al.^33^ introduced an enhanced shallow CNN (ES-CNN) for multi-classifying breast cancer histopathological images. There were three methods by which the research goals were met. Based on the findings from magnification and patient dependencies, we began by designing the architecture of the proposed network. Second, the author used the suggested network to build a multi-classification model. Third, the author ran trials with two different types of data: those measuring classification accuracy and those measuring computing use. The experimental findings showed that the suggested strategies outperform the current state of the art in classification with very little computational overhead. Using 400 ×, 200 ×, 100 ×, and 40 × image magnifications, respectively, this study reveals a multi-classification accuracy of 96%, 95%, 98%, and 96%.

Early identification is key to improving survival rates for breast cancer, making medical imaging a critical field of study. Support vector machines (SVMs), decision trees, and k-nearest neighbors (KNNs) are some traditional classification algorithms that have shown effectiveness in evaluating histopathological and mammography images. Inconsistent findings in diverse datasets are a common consequence of these approaches’ reliance on human feature extraction and inflexibility when dealing with complicated picture data. The capacity of CNNs to automatically build hierarchical representations has made them the gold standard for medical picture categorization since the advent of DL. Much notable research has shown that CNN versions, including VGGNet, AlexNet, and DenseNet, perform well in breast cancer classification tasks. On the other hand, these designs may have issues with disappearing gradients, sluggish convergence, and an excessive need for labeled data during training. Introduced ResNet models use skip connections to offer deeper topologies with enhanced gradient flow and efficiency, addressing some of these constraints. Even with these improvements, Raw CNN-based models struggle to differentiate between malignant tissue structures with fine detail, particularly in low-contrast or noisy pictures. This led to the development of hybrid strategies. Researchers have used wavelet transformations to reduce noise and extract features at several scales. To fine-tune the parameters of the network, they used optimization methods such as particle swarm optimization (PSO) and genetic algorithms (GA). Although there is potential in these methods, they often use heuristic optimization procedures and fixed scoring processes, which may not work so well with changing picture attributes. Medical image processing has lately become interested in fuzzy logic, which is renowned for dealing with ambiguity and uncertainty in decision-making. It makes it possible to score and understand picture elements with greater subtlety. Yet, there has been a dearth of research into developing a cohesive framework for breast cancer diagnostics that effectively combines fuzzy logic with deep CNNs. Additionally, the inefficiency of current optimization methods in real-time medical applications is due to their lack of flexibility. While previous research has made strides in breast cancer classification using hybrid and DL approaches, there is still a significant lack of a unified model that combines adaptive optimization, fuzzy scoring, and multi-resolution feature extraction. Research on efficient, reliable, and resilient breast cancer diagnosis using ResNet-based CNNs with advanced approaches such as the Adaptive Grey Wolf Optimization Algorithm (AGWOA) and Fast Discrete Wavelet Transform (FDWT) is lacking.

## Proposed methodology

**Fig. 1:**
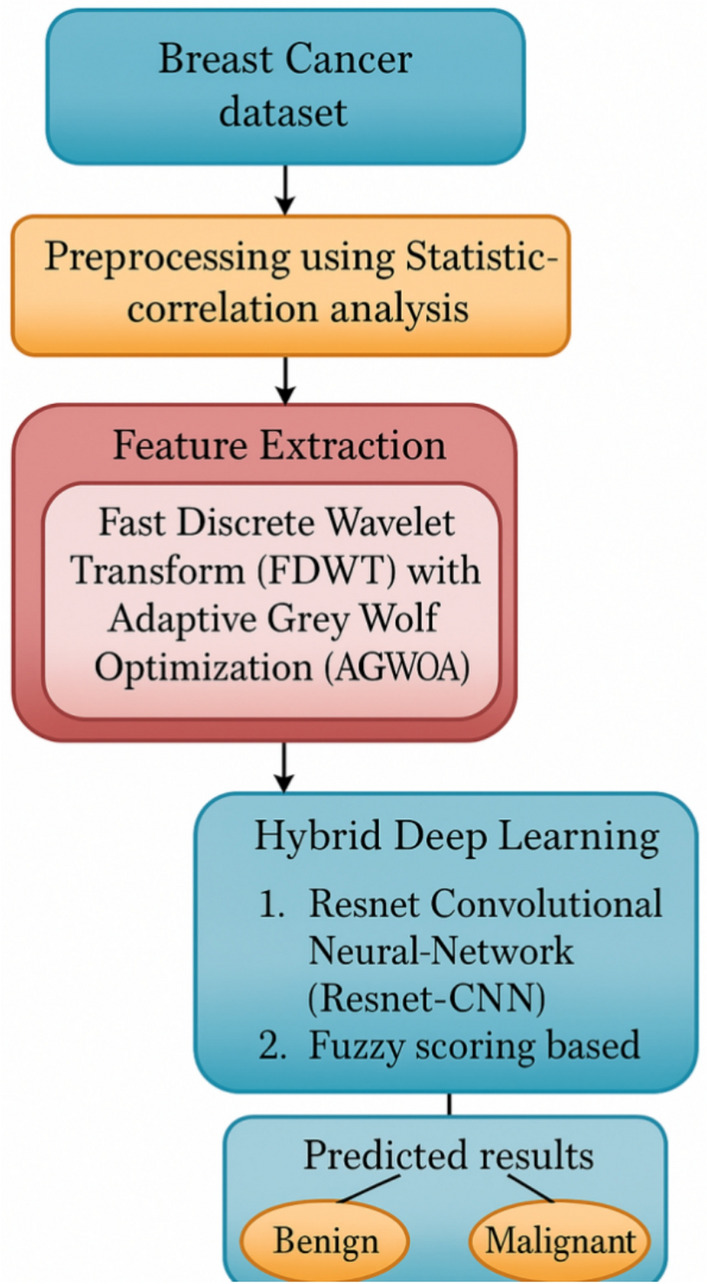
Procedure of the breast cancer classification.

The first step involved in this methodology is Image Acquisition. The image acquisition has been performed in the proposed work. The input images are initially acquired from the publicly available Break His database. Once the data has been collected, the next stage is executed to classify breast cancer.This work first introduces a pre-processing method based on statistical correlation analysis to increase the classifier’s effectiveness. The features are extracted from the ROI images using the wrapping technique and anFDWT.Both the extracted curvelet coefficients and the turn-time difficulty are excessive for classification.The adaptive grey wolf optimization technique was presented using swarm intelligence to minimize the time difficulty and choose key characteristics. It presents the FS-Resnet CNN approach, a revolutionary building component for network optimization. In addition, the suggested FS-Resnet CNN framework preserves memory effectively, is less susceptible to noise, and is computationally effective. Figure 1. Illustrate the procedure of the present methodology.All images were resized to a consistent dimension of 224 × 224 pixels to align with the FS-ResNet CNN input requirement. Then, color normalization using the Reinhard method was applied to mitigate slide staining variability. This statistical normalization ensures that all input data follow a zero-mean, unit-variance distribution, accelerating convergence and stabilizing gradient flow during model training.

### Pre-processing using statistical correlation coefficient (Scc)

The statistical correlation coefficient-based pre-processing method is initially used to process the breast cancer dataset.The intensity of the relationship between the two parameters and the mean of all the values of the second factor that correspond to the dependent, response, result, and match is known as the correlation^21^. When x is a random covariate to y, i.e., when x and y change together, the sample correlation coefficient, rxy(r), is the statistical measure used to evaluate the power of the linear relationship instead of making predictions. Numerous numerical techniques are available to assess how fine a regression equation fits the data, in addition to charting. The sample coefficients of determination (R^2^) for the linear regression f are a helpful verification tool. For a framework with a constant term (w_i_ = 1),R^2^ is the rate of the number of squares included by the regression ${SS}_{Reg}$ to the overall number of squares of variation regarding the mean ${S}_{YY}$.

1 $${R}^{2}=\frac{{SS}_{Reg}}{{S}_{YY}}=\frac{{S}_{YY}-SSE}{{S}_{YY}}=1-\frac{SSE}{{S}_{YY}}=1-\frac{{({y}_{i}-\widehat{{y}_{i}})}^{2}}{{({y}_{i}-\overline{{y }_{i}})}^{2}}$$

where $\widehat{y}$ indicate the *y ‘s* expected value and $\overline{y }$ is the mean of *y’s* value; either summation is over *i* = 1,2,…, n. *SSE* is the remaining sum of squares. *R*^*2*^ = 1 − *SSE/SST* in a simulation without a constant term, wherein SST is the sum of the squaresof *y*^2^. The percentage of the total variance in the mean $\overline{y }$ that the regression explains is shown by *R*^2^ in Eq. (1). Therefore, the more *R*^2^ there is, the less the introduction of the independent variable *x* will change the overall variance of $\overline{y }$. A common method of expressing it as a percentage is to multiply it by 100. Since 0 ≤ *SSE* ≤ *SS*_*YY*_, it follows that 0 ≤ *R*^2^ ≤ 1. Actually, *y* and $\widehat{y}$ correlate R.

2 $$R={r}_{y\widehat{y}}=\frac{{\sum \left({y}_{i}-\widehat{{y}_{i}}\right)(y}_{i}-\overline{{y }_{i}})}{[{{\sum \left({y}_{i}-\widehat{{y}_{i}}\right)] [(y}_{i}-\overline{{y }_{i}})]}^{1/2}}$$

It’s sometimes referred to as the multiple correlation coefficients. Comparing the *R*^2^ of two equations with varying amounts of coefficients that were created utilizing the same data set is inappropriate. Despite that, looking at *R*^2^ in a regression output is a helpful feature. To clarify the notation, the coefficient of determination in a straightforward regression with a constant component equals the square of the correlation coefficients between x and y.

3 $${r}_{xy}=\pm \sqrt{{R}^{2}=\sqrt{1-\frac{{S}_{YY}-{a}_{1}^{2}{S}_{XX}}{{S}_{YY}}}}={a}_{1}\sqrt{\frac{{S}_{XX}}{{S}_{YY}}}=\frac{{S}_{XY}}{\sqrt{{{S}_{XX}S}_{YY}}}$$

Depending on the slope, a1 is either positive or negative for the fitted regression line, and/or minus signsare assigned to this metric. If*R*^2^ equals unity, every variance can be clarified, and the regression line represents a perfect match for every point.The regression linesare horizontal, and y is not a function of x if the coefficientsare zero, meaning that the line explains nothing.Regression coefficients in additional generic regression situations are more intricately correlated with a correlation of the kind *r*_*xy*_.

The covariance among the two random parameters, *x* and *y,* is the estimated cost of the sum of their deviations from their expected values. It is a measure of how two quantities of fluctuation are correlated. The sample covariance that is presented is.

4 $$cov\left(x,y\right)=\frac{1}{n-1}\sum {(x}_{i}-\overline{x }){(y}_{i}-\overline{y })$$

5 $$cov\left(x,y\right)\le {s}_{x}{s}_{y}$$

This recommends immediately that r ≤ 1.The covariance measures correlations between x and y. If the two factors have a linear relationship, the covariance will either be negative or positive and will be created on the slope of the relationship. The covariance is 0 if *x* and *y* are autonomous, that is, not connected. The opposite isn’t always true, as examples of extremely dependent random variables with zero covariance can be created frequently nonlinearly.

The variance is a particular instance of a random variable’s covariance with itself, even though covariance is frequently disregarded in beginning textbooks.The standard deviations, represented by σ for the population and s for samples, are the square root of the variance and are always positive. At the very least, covariance must be considered when realistic uncertain points are present Table 2 shows the nomenclature.

**Table 2:** Nomenclature.

Variable	Description
${SS}_{Reg}$	Squares included by the regression
${S}_{YY}$	Squares of variation regarding the mean
$SSE$	The remaining sum of squares
$y$	Summation value
$\widehat{y}$	Expected value
${R}^{2}$	Coefficients of determination
$x and y$	Random covariate
$cov\left(x,y\right)$	Covariance measures
$\varphi \left(f\right)$	function of frequency
$\varphi (t)$	Fourier transform
$J$	J-level wavelet decomposition
$f\left(t\right)$	Time series information or functions
${S}_{j}\left(t\right)$	Smooth coefficients
${D}_{j}\left(t\right)$	Detailed coefficients
$\overrightarrow{{X}_{p}}$	prey’s position vector
$N$	Pareto optimum solution
$c$	Constant number
$F\left(x, \left\{{W}_{i}\right\}\right)$	Residual mapping
$\sigma$	Sigmoid Function
$F + x$	Element-wise additions
${W}_{s}$	Linear projection
$E\left({y}_{i}{x}_{i}\right)$	Anticipation
${W}_{i}$	Weight
${b}_{i}$	Bias

### Feature extraction utilizingFDWT

The feature extraction process is utilized after pre-processing the given breast cancer data.The wrapping technique and FDWT are attributes of the ROI mammography images. The retrieved wavelet coefficients and the run-time difficulty are too high to be classified.Swarm intelligence was utilized to alter the whale optimization algorithm to reduce complexity in time and choose the salient features.

#### Fast discrete wavelet transform (FDWT)

An FDWT is a transform that divides a provideddata set into numerous sets, eachwith a temporal sequence of coefficients showing the information’s evolution in the corresponding frequency band over time^22^. As discussed, the wavelet transform is the statistical paradigm for converting the raw observation into time-scale domains. Most data is non-stationary, so the WT-oriented framework works well for data analysis. The DWT comprises the maximum overlapping wavelet transforms, Daubechies, Haar, and other functions. With varying uses, each of these functions has the same characteristics. Wavelet theory represents the Fourier approach for any role as the product of the sine and cosine functions.A fundamental notion is that a wavelet is only a function of time t that satisfies the wavelet admissibility conditions.

6 $${C}_{\varphi }={\int }_{0}^{\infty }\frac{\left|\varphi (f)\right|}{f}df<\infty$$

wherein $\varphi (f)$ denotes a function of frequency $f$ and $\varphi (t)$ is the Fourier transform. An approach called the WT may be utilized to extract features from the images of diabetic retinopathy. It was developed to solve Fourier transform-related problems with non-stationary information localized in place, time, or frequency.

Two types of wavelets can be found in a function or family. Father wavelets describe a signal’s low-frequency, smooth components, but mother wavelets explain its high-frequency, complicated characteristics. The J-level wavelet decomposition has $j=\text{1,2},3,..., J$ representing the mother and father wavelets, respectively, represented by Eq. (7).

7 $${\varnothing }_{j,k}={2}^{\left(\frac{-j}{2}\right)}\varnothing \left(t-\frac{{2}^{j}k}{{2}^{j}}\right)$$

8 $${\varphi }_{j,k}={2}^{\left(\frac{-j}{2}\right)}\varphi \left(t-\frac{{2}^{j}k}{{2}^{j}}\right)$$

wherein $J$ stands for the greatest scale that can be achieved with the given quantity of data points and the two wavelet types (mother and father), and it meets the following:

9 $$\int \varnothing \left(t\right)dt=1and\int \varphi \left(t\right)dt=0$$

Wavelet analysis might be utilized for expressing time series information or functions $f(t)$ as an input. It is created as a series of projections onto mother and father wavelets, which are classified by $\{k\}, k = \{0, 1, 2,...\}$ and by $\{S\}=2j, \{j=\text{1,2},3,. .J\}$. Creating a lattice is necessary for computations when examining actual discretely sampled data. From a mathematical perspective, a dyadic expansion makes sense, as Eq. (10) illustrates. The following projections provide the expansion coefficients:

10 $${S}_{j,k}=\int {\varnothing }_{j,k}f\left(t\right)dt,\int {\varphi }_{j,k}f\left(t\right)dt,$$

11 $${S}_{j}\left(t\right)=\sum {S}_{j,k}{\varnothing }_{j,k}\left(t\right)and{D}_{j}\left(t\right)=\sum {S}_{j,k}{\varnothing }_{j,k}\left(t\right)$$

Equation (11) utilizes the WT to compute the value of the wavelet series approximation for a given set of data, where the smooth and detailed coefficients are introduced by ${S}_{j}\left(t\right)$ and ${D}_{j}\left(t\right)$, accordingly. The detailed coefficients are employed to identify the primary characteristics in the dataset, whereas the smooth coefficient extracts the significant features from the dataset. The run-time difficulty and the extracted wavelet coefficient are too great to be characterized. Swarm intelligence was utilized in the adaptive grey wolf optimization method to reduce time complexity and choose the salient features.

#### Grey wolf optimization (Gwo) 

This work presents the Adaptive Grey Wolf Optimizer (AGWO), a revolutionary optimization technique founded on the GWO.The primary sources of inspiration for this system were the social pyramid and hunting approaches of grey wolves. When creating GWO, the alpha (α) wolf is determined by selecting the solution that best fits the social hierarchy of wolves. Consequently, beta (β) and delta (δ) wolves, accordingly, are the terms of the 2^nd^ and 3^rd^ finest solution^23^. It is believed that the residual solution is omega (ω) wolves. The GWO approach uses α, β, and δ to direct its hunting. These 3 wolves are surveyed by the ω wolves as they look for the optimal. The subsequent equations were added to the social leadership to replicate the surrounding conduct of grey wolves during a hunt.Group hunting and their social grading are intriguing social characteristics of grey wolves. The following are the primary phases:

- Tracking, pursuing, and getting close to the target;
- encircling, pursuing, and bothering the target till it gives up;
- attacking the target

**Fig. 2:**
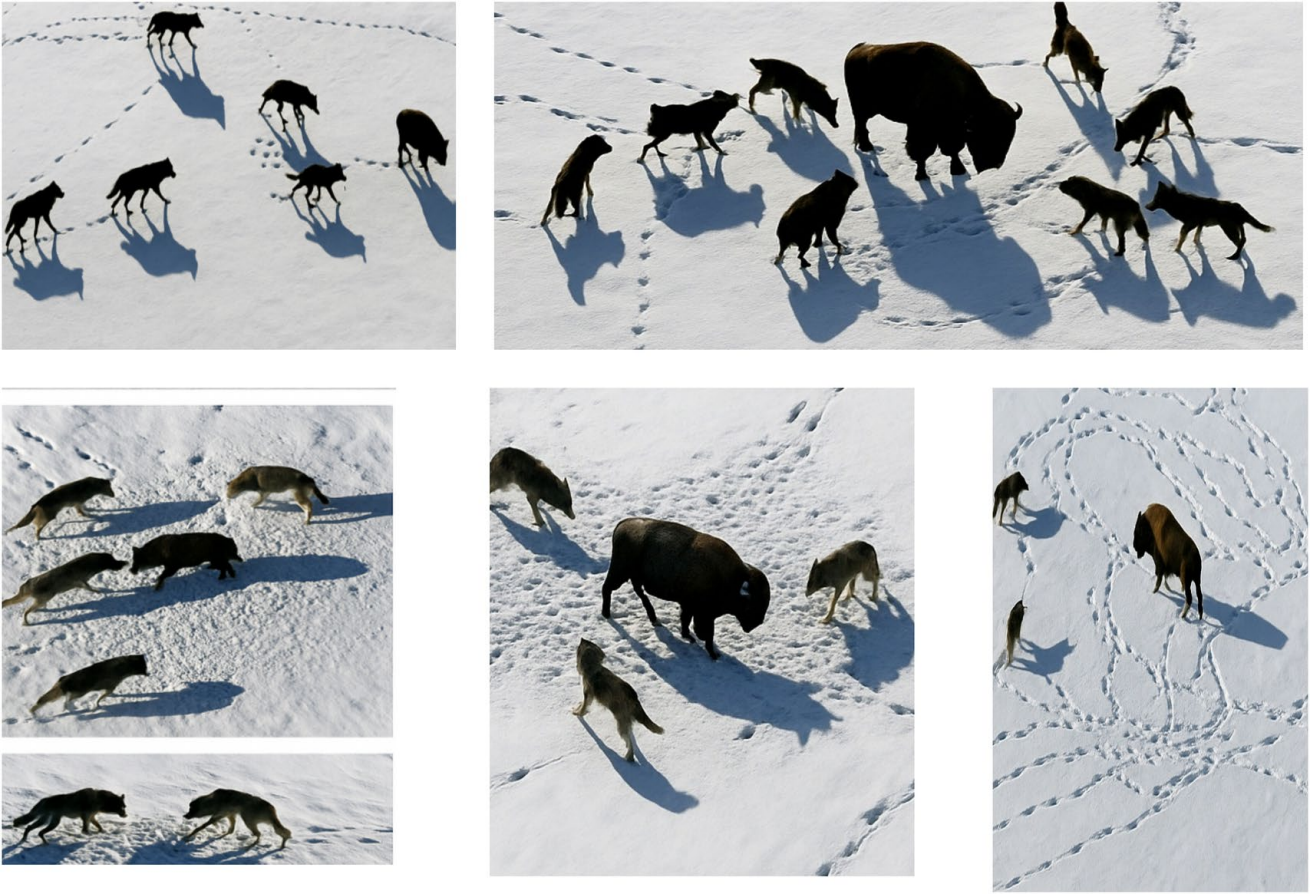
Grey WolvesHuntingBehavior: (**A**) Approaching, Chasing, and Tracking Prey (**B-D**) Harassing, Pursuing, and Encircling (**E**) Stationary Situation and Attacks^27^. The dataset link for Fig. 2 is attached in the reference.

These actions are depicted in Fig. 2.

### Statistical model and algorithms

The representations of tracking, encircling, attacking, and social hierarchy are given in this chapter. The GWO method is described.

#### Social pyramid

Use alpha $\alpha$, to characterize the social structure of the wolves while developing GWO. Therefore, beta $\beta$ and delta $\delta$ stand for the 2^nd^ and 3^rd^-best answers, correspondingly. The residual potential answers are anticipated to be omega $\omega$. $\alpha$, $\beta$, and $\delta$ to guide the hunting process in the GWO system. Out of all the wolves, these three are the leaders.

#### Encircling prey

Grey wolves encircle their victim during a hunt, as was earlier established. To represent encircling behavior analytically, suggested:

12 $$\overrightarrow{D}=\left|\overrightarrow{C}.\overrightarrow{{X}_{p}}\left(t\right)-\overrightarrow{X}(t)\right|$$

13 $$\overrightarrow{X}\left(t+1\right)=\overrightarrow{{X}_{p}}\left(t\right)-\overrightarrow{A}.\overrightarrow{D}$$

where $\overrightarrow{{X}_{p}}$ represents the prey’s position vector and signifies the position vectors of a grey wolf, and *t* is the present iteration and coefficient vectors. Here is how the vectors are computed:

14 $$\overrightarrow{A}=2\overrightarrow{a}.\overrightarrow{{r}_{1}}-\overrightarrow{a}$$

15 $$\overrightarrow{C}=2.\overrightarrow{{r}_{2}}$$

**Fig. 3:**
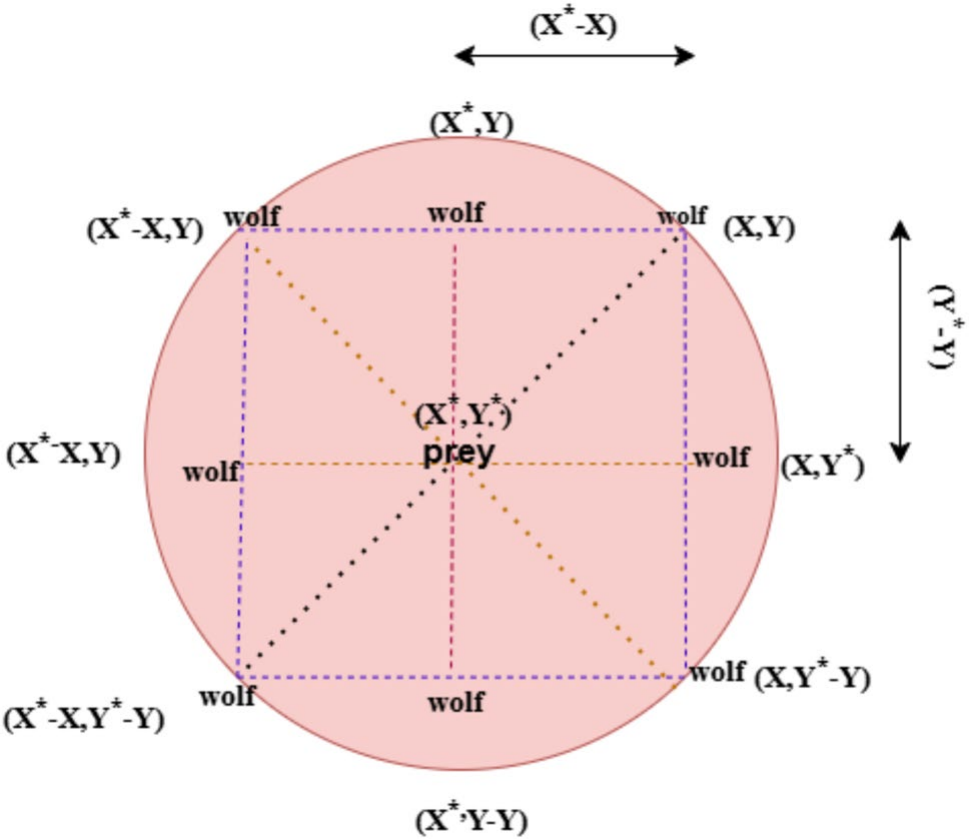
Location vectors and their achievable next positions.

When the variables are linearly lowered from 2 to 0 across the iterationand are random vectors in [0,1], in Fig. 3, some possible neighbors and a 2D position vectorillustrate Eqs. (14) and (15).This diagram illustrates how the positions of the prey (X*,Y*) can affect a grey wolf in the position of (X,Y).By changing the value ofvectors, numerous locations around the ideal agent can be reached, depending on the current location.For instance, (X*X,Y*) is obtained through the setting.Note that because of the random vectors, the wolves can go to any place among the locations displayed in Fig. 3.Therefore, grey wolves can change their location within the area around the prey at any random spot by utilizing Eqs. (14) and (15).

Using the same general idea, the gray wolf will travel in hyper-cubes within the best solution found thus far in an n-dimensional search space.

#### Hunting

Grey wolves possess the capability to find and loop around their prey. The alpha typically leads the hunt. From time to time, the delta and beta could go hunting. However, it’s unclear where the ideal position is in abstract search spaces. To statistically replicate the hunting behaviors of the grey wolf, presume that the beta, delta, and alpha have more data about the possible locations of prey. Thus, the top three results have been maintained, and the other search agents have been mandated to control their locationon the top search agent’s page. In this context, the equations are suggested:

16 $$\overrightarrow{{D}_{\alpha }}=\left|\overrightarrow{{C}_{1}.}\overrightarrow{{X}_{\alpha }}-\overrightarrow{X}\right|, \overrightarrow{{D}_{\beta }}=\left|\overrightarrow{{C}_{2}.}\overrightarrow{{X}_{\beta }}-\overrightarrow{X}\right|, \overrightarrow{{D}_{\delta }}=\left|\overrightarrow{{C}_{3}.}\overrightarrow{{X}_{\delta }}-\overrightarrow{X}\right|$$

17 $$\overrightarrow{X}\left(t+1\right)=\frac{\overrightarrow{{X}_{1}}+\overrightarrow{{X}_{2}}+\overrightarrow{{X}_{3}}}{3}$$

#### Attacking prey (exploitation)

**Fig. 4:**
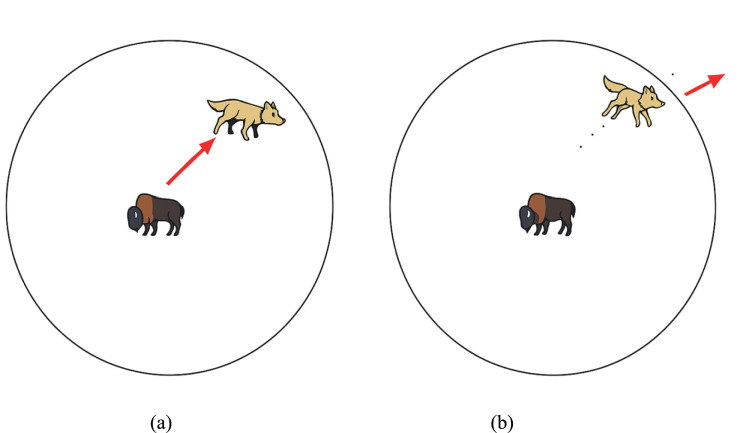
Attacking prey Vs. searching for prey.

As mentioned, the grey wolves finish their hunt by charging at their victim the second it stops moving. Downplay the importance of detailing the mathematical hunt for the quarry. Unless otherwise specified, this is a random value between -a and a, where a falls between 2 and 0 as the number of repetitions increases.The search agent’s future location can be anywhere between its present location and the prey’s location when random values fall within the range[−1,1]when |A|< 1,the wolves are compelled to attack toward the direction of the prey,as seen in Fig. 4(a).s

By applying the operators proposed so far, the search agents of the GWO system may update their locations concerning the beta, alpha, and delta locations and then assault their prey. Using these operators, the GWO approach often gets stuck in local solutions. The proposed encircling system shows signs of exploration, but GWO needs additional operators to prioritize exploration.

#### Search for prey (exploration)

Grey wolves primarily utilize the beta, alpha, and delta positions to guide their searches. When hunting, they separate from one another and reunite when attacking. To imitate divergence, utilize random values superior to or equal to −1 to induce the search agent to separate from the prey. The examination is prioritized, and the global search capability of the GWO method is enabled. Furthermore, as seen in Fig. 4(b), grey wolves must deviate from their prey to locate a more suitable meal when |A|> 1. Another feature of GWO is that it promotes exploration. Equation (5) shows that the values of the vector in [0, 2] are random. This component can stochastically increase (*C* > *1*) or decrease (*C* < *1*) the contribution of prey to the distance in Eq. (2). As a result, GWO can behave more erratically throughout optimization, promoting discovery and preventing local optima. Notably, C deviates from A in that it does not decrease linearly. Consciously ensure that C returns a random result to promote exploration in the first and final rounds. If the local optimum becomes stuck, this component may help, especially in the final few iterations. The C vector could be considered the effect of natural obstacles that hinder prey from entering. The ability of wolves to quickly and effectively catch prey is hindered by the natural obstacles they face on their hunting paths. The vector C achieves this exact result. Wolf packs are notoriously hard to locate because the wolf’s propensity to add weight to its prey randomly makes hunting in such dense areas very challenging.Reducing the value from 2 to 0 highlights exploration and exploitation. It is common for candidate responses to veer away from the prey when the absolute value of || is more than 1 and towards the prey when absolutely zero.Ultimately, the fulfillment of an end criterion ends the GWO method.

### Adaptive grey wolf optimization

The GWO method begins to be exploited when *|A|*< *1*. An investigated agent’s location agent liesbetween its current location and the prey’s location, where random values of *A⃗* are in [−1,1], which aid the search agents converging around a predicted prey position supplied by alpha, beta, and delta answers.The GWO technique generates an array of random answers called the initial population before beginning optimization. The three most excellent solutions found currently are retained and referred to as alpha, beta, and delta solutions throughout optimization. Positions are triggered for each omega wolf. During the repetition, variables a and *A* drop linearly. Thus, when |*A⃗*|> 1, search agents typically diverge from the prey; conversely, when |*A⃗*|< 1, they converge closer to the prey. When an end condition is satisfied, the alpha solution’s location, score, and the best answers found during optimization are ultimately returned. Merge two new elements that enable multi-objective optimization by GWO. The parts that are used are remarkably identical. First of all, there is an archive that houses all of the previously found non-dominated Pareto optimum solutions. The second part is a strategy for selecting leaders from the archive, which helps identify alpha, beta, and delta options as the primary focus of the hunt. The archive is a straightforward storage device that can store and retrieve previously found non-dominated Pareto optimum solutions.

The quantity of options in the hypercube increases the possibility of eliminating a solution. If there is too much data in the archive, the crowded sections are chosen first, and a solution is randomly detached from one of them to produce room for an alternative. A unique situation exists when solutions are added outside of the hypercubes. In this instance, each component is expanded to encompass the novel solutions. Thus, the components of other solutions also change. The procedure for choosing the leader is the second element. To locate a solution near the global optimum, these leaders direct the remaining search agents onto interesting areas of the search space. However, in multi-objective search spaces, the principles of Pareto optimality covered in the previous part make it difficult to assess the answers. This problem is described by the method for electing leaders. As was already indicated, the finest non-dominated outcomes found to date are archived. Beta, alpha, or delta wolves are examples of non-dominated solutionspresented by the leader election element, which selects the smallest populated areas of the search space. Utilizing a roulette-wheel technique, a particular probability is assigned to each hypercube for choosing:

18 $${P}_{i}=\frac{c}{{N}_{i}}$$

As shown in Eq. (18), *N* is the quantity of the Pareto optimum solution found in the *i*-th segment, and *c* is a constant number larger than 1.Less populated hypercubes have a better chance of recommending new leaders, according to Eq. (18). As the quantity of determined solutions in the hypercubes decreases, the likelihood of choosing hypercubes to elect leaders from rises. Note that in certain exceptional circumstances, selecting three leaders may be necessary. Three of these are given randomly to three solutions if three are in the least packed section. It has been discovered that if the first least crowded hypercubes have fewer than three solutions, more leaders can be chosen from the second least crowded hypercube.The issue remains the same if the second least packed hypercube has one solution; hence, the third least congested hypercube must be used to pick the delta leader. This method stops AGWO from choosing leaders with alpha, beta, or delta traits. If there are insufficient leaders in the least populated sector, the leader election technique prioritizes the least packed hypercubes and picks leaders from multiple places, forcing the search to concentrate on the unexposed portions of the search space.Algorithm 1 shows the pseudocode of the suggested adaptive GWO.

**Algorithm 1:**
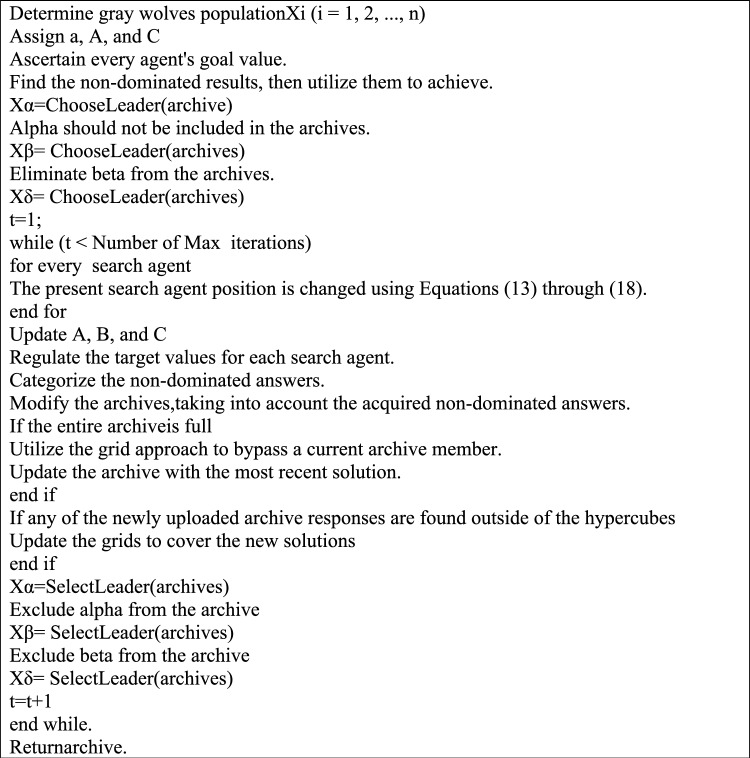
Pseudocode of suggested adaptive GWO.

### Hybrid deep learning

Create a learning task for classifying breast cancer using the multi-task learning framework because some breasts have both benign and malignant features. Specifically, designate two binary labels for every breast: whether malignant findings are present or absent, and whether benign findings are present or absent. Utilize the multi-task educational structure to create breast cancer categorization as a learning task, maintaining that some breasts include both benign and malignant results. It should designate two binary labels for every breast: the existence or absence of malignant or benign results. A degradation issue was discovered when deeper networks could begin converging: accuracy rises with network depth until it becomes saturated and rapidly declines. The findings verify that adding additional layers to a sufficiently complex structure increases training error, which is interestingly not driven by overfitting. The degradation shows that not all systems can be optimized with the same ease. Let’s examine two types of architecture: one shallower and the other deeper, with more levels. An architectural solution to the complicated structure is available: the extra layers are determinedby mapping, and the remaining layers are replicas of the obtained shallower form. Training errors for a shallower model shouldn’t be higher than those for an extended model because of this artificial solution. Research shows, however, that existing solvers cannot find alternatives that are just as good as the original plan.A deep residual learning architecture to challenge the degradation issue. The network consists of one average pooling layer after three convolutional layers.

Every layer’s outputs serve as the inputs for the layers that follow. As illustrated in Fig. 2b, the structure includes a single, completely interconnected cascaded residual block. All of the preceding convolutional layers’ inputs are fed into each convolutional layer in the residual network. Three convolutional layers are the most effective for this framework, according to the empirical quantity of convolutional layers that have been established. Before the data is fed to the classifier, the convolutional layers execute convolution operations, and the final pooling layer performs average pooling operations. Rather than the standard sequential method, the very complicated Resnet architecture uses a network-in-network layer, a pooling layer, big and small convolutional layers meant to operate in parallel, and so on.Thisresearch presents an innovative building block called the FS-Resnet CNN approach to improve this network, which offers faster convergence than the traditional stochastic gradient descent method. Additionally, the suggested FS-Resnet CNN paradigm utilizing fuzzy scoring is memory-efficient, less susceptible to noise, and cost-effective.

#### Residual (ResNet) learning model

**Fig. 5:**
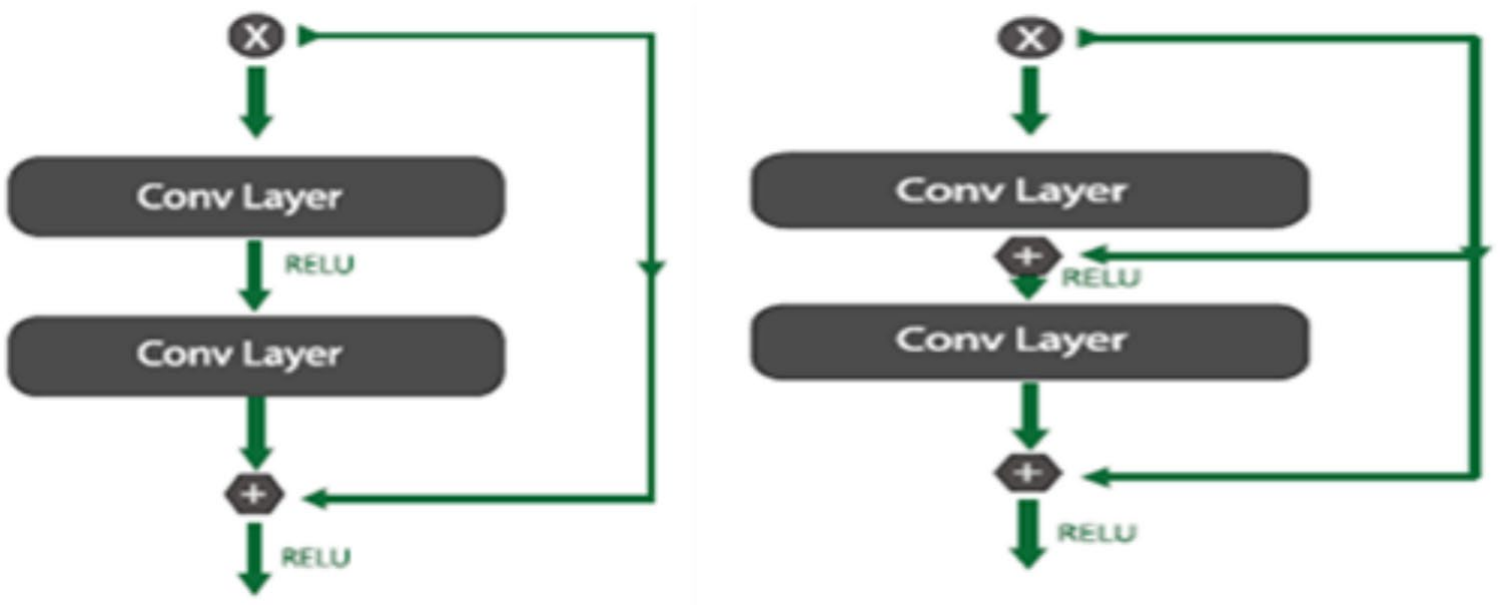
(**A**). Core building block of deep residual networks. (**B**). Modified version of the residual block: With fully connected cascaded layers.

The study proposes a ResNet-CNN framework that adopts and combines the essential concepts of the traditional CNN and the ResNet models to maximize their respective benefits. The concepts of CNN and ResNet have demonstrated the capacity to scale up numerous layers while maintaining increased effectiveness and performance. Numerous residual blocks with identity mapping make up the ResNet. Multiple convolutional networks comprise the deep convolutional network’s CNN framework^24^. A CNN model typically includes one or more fully connected (FC) layers after many convolutional and subsampling layers. Multilayer neural networks often use the FC layer as an example. Downsampling data is the responsibility of the pooling layer, while the convolutional layer compresses the input picture using several filters. The pooling layer has two characteristics: maximum pooling and average pooling. CNNs use stacked layers to transform input images from their original raw pixel composition to their final class scores.CNN structures have served as foundational elements for multiple theories of semantic segmentation. ResNet and CNN are strong, sophisticated DL simulations. The central component of the ResNet is depicted in Fig. 5.

As demonstrated in Fig. 5a, the network has 152 layers and an innovative strategy that improves residual blocks to solve the problem of training a complex structure with identity skip connections. The inputs from the layers are copied by these leftover blocks and forwarded to the subsequent layer. The unique skip connection step ensures that a subsequent layer may acquire something new from the input it is currently familiar with, which solves the problem of disappearing gradients.

#### Residual representation

**Fig. 6:**
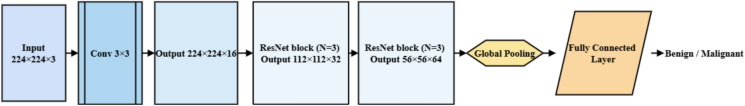
Architecture of proposed ResNet-CNN model.

Allow x to input the first of these layers, and let $H(x)$ be the basic mapping to matcha few stacked layers. Provided that the input and output have the same parameters, the hypothesis that several non-linear layers can asymptotically approach complex functions is equal to the hypothesis that they asymptotically equal the remaining functions, or *H*(x) − x. Hence, let the stacked layers explicitly method a remaining function $F(x) = H(x) - x$ rather than expecting them to resemble *H*(x). As an outcome, the actual function yields $F(x)+x$. The simplicity of learning for each form may differ, even supposing both ought to be able to estimate the target function asynchronously. The surprising phenomenon regarding the deterioration issue (Fig. 5.a, left) is the motivation behind this reformulation. As mentioned, if the extra layers are created as identity mappings, a deeper framework has a lower training error than its shallower equivalent. Approximating comparable mappings by several non-linear layers may be problematic for solvers due to the degradation issue. Using the residual learning reformulation, the solvers may obtain optimum identity mappings by reducing the weights of the various non-linear layers to zero. While identity mappings may not be the best choice in practical settings, this revised approach might assist with problem preconditioning. Instead of learning the function as a new one, the solver could discover the perturbations more easily if the ideal function is more like the same mapping than a zero mapping, as compared with an identity mapping. Based on the research, identity mappings seemto be an appropriate preconditioning since learned residual functions usually produce tiny responses (Fig. 6).Additionally, add residual learning to each stack of a few levels. In Fig. 6, a building block is displayed. In formal terms, a building block is described as:

19 $$y=F\left(x,\left\{{W}_{i}\right\}\right)+x$$

Thus, x and y represent the layers under consideration’s input and output vectors. To be obtained, the residual mapping is signified by the functions*F*(x*, {Wi}*). For the 2-layerinstance in Fig. 6, *F* = *W*2*σ*(*W*1x), whereas *σ* signifies ReLUs, and the bias is removed to simplify notation.

The operations $F + x$ are completed using element-wise additions and shortcut connections. Following the addition, the 2^nd^ non-linearity will be implemented. The shortcut associates in Eqnpresent no additional variable. (6) This is significant for the comparisons among plain and remaining networks, in addition to being required in practice. And fairly evaluate the plain/residual networks with the same computing expense, depth, width, and quantity of variables simultaneously. In Eq. (20), the dimensions of x and *F* have to be similar. If not, use the shortcut links to accomplish a linear projection *Ws* that complies with the dimensions:

20 $$y=F\left(x,\left\{{W}_{i}\right\}\right)+{W}_{s}x$$

Apply *Ws*, a square matrix, to Eq. (20). However, investigations show that *Ws* is employed when matching dimensions and that the identification mapping is inexpensive and adequate for resolving the degradation issue. The remainingfunction, *F,* has flexible shapes. The function F with two or three layers was utilized in this research testing (Fig. 3),while more layers might be used. Nevertheless, Eq. (6) resembles a linear layer $(y=w1x+x)$ if F is a single-layer system, as no advantages were observed. It should be emphasized that convolution layers can benefit from the previously specified notations, even if they are simplified to fully connected layers. Multiple convolution layers are signified by the functions F(x, {Wi}, channel-by-channel, and element-wise additions on 2 feature maps.

#### Shortcut connections

Theoretical and practical approaches that result in shortcut connections have long been researched. In the initial stages of MLP training, a linear layer is added to link the network’s input to its output. To handle vanishing/exploding gradients, a few intermediary layers are directly linked to auxiliary classifiers. Shortcut connections implement gradients, transmitted errors, and centering layer replies.

A shortcut and a few deeper branches constitute a“ResNet”layer.“Highway networks”offer shortcut connections with gating functions concurrently with this operation. Unlike the parameter-free identity shortcuts, these gates utilize data and have variables. The highway network layers demonstrate non-residual functionality if a gated route is"closed."The formulation always teaches remaining functions, identifying closed shortcuts and all data that are forever passed along with more residual functions to be acquired. Highway networks haven’t verified any improvement in accuracy at very deep depths.The ResNet is a high-complexity, 22-layer design that utilizes a network-in-network layer, pooling layers, and a big and tiny convolution layercomputed in parallel instead of the usual sequential approach. To optimize our network, this work presents a novel building block called the Fuzzy Scoring Resnet- CNN (FS-Resnet CNN) model, which offers faster convergence than the traditional stochastic gradient descent. Additionally, the suggested Fuzzy Scoring based Resnet-CNN (FS-Resnet CNN) model is memory-efficient, computationally efficient, and less susceptible to noise.

## Fuzzy scoring and structure of fuzzy fully connected layers

Assume training setsare $T=\left[\left({x}_{1},{y}_{1}\right),\left({x}_{2},{y}_{2}\right),\dots \dots .,\left({x}_{n},{y}_{n}\right)\right],$ whereas ${x}_{i}$ is the parameter conveying the data, and ${y}_{i}$ is related labels for all *i* = 1,2,…,*n*, whereas *n* is the number of training samples^24^. Considering that the samples were divided into*m*-scoring groups, each of which represents the actual score $S=\left[{S}_{1},{S}_{2},...,{S}_{m}\right]$ for a more precise assessment, the projected score *S̃* = [*S̃*1,*S̃*2,…,*S̃m*] is precede by decimal parts.

Fuzzy Function: Utilizing directed extensional scores, this fuzzy function reduces the effect when comparing relative classes. In CNNs, the sigmoid function defines the probabilities:

21 $$P\left({x}_{i}|{y}_{i}=i\right)=\frac{{e}^{-E({y}_{i}{x}_{i})}}{{\sum }_{{y}_{1}}^{{y}_{m}}{e}^{-E({y}_{i}{x}_{i})}}$$

Together with its neighbors to the left and right:

22 $$P\left({x}_{i\pm 1}|{y}_{i\pm 1}=i\pm 1\right)=\frac{{e}^{-E({y}_{i\pm 1}{x}_{i\pm 1})}}{{\sum }_{{y}_{1}}^{{y}_{m}}{e}^{-E({y}_{i\pm 1}{x}_{i\pm 1})}}$$

where $E({y}_{i}{x}_{i})$ is the anticipation that ${x}_{i}$ is forecast as ${y}_{i}$, and *m* is the quantity of the classes.Depending on specific previous popular research on CNNs, there are numerous affine forms for both the $(xi|yi)\euro \left( {0,1} \right)$ and this probabilistic distribution.

23 $$P\left({x}_{i}|{y}_{i}=i\right)=\sigma ({W}_{i}{x}_{i}+{b}_{i})$$

24 $$P\left({x}_{i\pm 1}|{y}_{i\pm 1}=i\pm 1\right)=\sigma ({W}_{i\pm 1}{x}_{i\pm 1}+{b}_{i\pm 1})$$

where ${W}_{i}$ isthe layer *i*’s weight and ${b}_{i}$ is the layer *i*’s bias. The recursive score was determined to lessen the conglutination among two neighboring or distant classes.

25 $$\widetilde{{V}_{0}}=\frac{i{\sum }_{i-a}^{i+b}P\left({x}_{i}|{y}_{i}\right)}{{\sum }_{i-a}^{i+b}P\left({x}_{i}|{y}_{i}\right)}$$

If *a* denotes a negative growth tendency and *b* denotes a positive increasing trend, for instance, *a* represents the aberrant trend toward cancer and the normal trend toward health. Equation (19)‘s redistributed probabilities were optimized, and as a result, the output values changed by the aforementioned operator tended to advance to the global average position.The fuzzy scoring mechanism addresses inherent uncertainty and ambiguity in histopathological features, particularly at tissue boundaries and regions with mixed malignancy patterns. Traditional crisp scoring functions in CNNs treat pixel-level or patch-level activations as binary or sharply weighted contributions, which can result in suboptimal learning from borderline or noisy regions. By contrast, the fuzzy scoring approach applies a rule-based membership function that assigns confidence weights based on the degree of feature activation rather than absolute values, thereby enabling more nuanced learning.

In this work, membership functions are implemented using Gaussian curves, whose parameters are initialized based on a statistical analysis of the training data distributions and subsequently fine-tuned via a grid search on a validation set. Class thresholds are dynamically adjusted during training to optimize the trade-off between sensitivity and specificity, ensuring robust handling of borderline cases.

26 $$\widetilde{P}\left({x}_{i}|{y}_{i}=i\right)=\frac{\left|i-{V}_{0}\right|}{b-a}\times {\sum }_{j=i-a}^{i+b}\frac{{e}^{-E({y}_{j},{x}_{j})}}{{\sum }_{{y}_{1}}^{{y}_{m}}{e}^{-E({y}_{j},{x}_{j})}}$$

Lastly, a modification was made to the backpropagation error across the estimation and the real data from

27 $${\varepsilon }_{i}={y}_{i}-P\left({x}_{i}|{y}_{i}=i\right)$$

to

28 $${\widetilde{\varepsilon }}_{i}={y}_{i}-\widetilde{P}\left({x}_{i}|{y}_{i}=i\right)$$

The impact of the altered ${\widetilde{\varepsilon }}_{i}$ computed:

29 $$\phi ={\widetilde{\varepsilon }}_{i}-{\varepsilon }_{i}$$

then,

30 $$\phi =\frac{\left|i-{V}_{0}\right|}{b-a}\times {\sum }_{j=i-a}^{i+b}\frac{{e}^{-E({y}_{j},{x}_{j})}}{{\sum }_{{y}_{1}}^{{y}_{m}}{e}^{-E({y}_{j},{x}_{j})}}-\frac{{e}^{-E({y}_{i},{x}_{i})}}{{\sum }_{{y}_{1}}^{{y}_{m}}{e}^{-E({y}_{i},{x}_{i})}}$$

Given that *a* and *b* are factors, it may be determined that the distance between *a* and *b* is constant. The left half of the formula ought to be utilized

31 $$\frac{\left|i-{V}_{0}\right|}{b-a}<1$$

If the probability in categories $i-a$ and $i+b$ are the same, it will be seen that:

32 $$\phi =\left(\frac{\left|i-{V}_{0}\right|}{b-a}-1\right)\times \frac{{e}^{-E({y}_{i},{x}_{i})}}{{\sum }_{{y}_{1}}^{{y}_{m}}{e}^{-E({y}_{i},{x}_{i})}}$$

Thus, $\phi \le 0$ identifying the category *i* as the highest across each group, even though probabilities from class $i-a$ to class $i+b$ are not necessarily equal:

33 $${\sum }_{j=i-a}^{i+b}\frac{{e}^{-E({y}_{j},{x}_{j})}}{{\sum }_{{y}_{1}}^{{y}_{m}}{e}^{-E({y}_{j},{x}_{j})}}\le \frac{{e}^{-E({y}_{i},{x}_{i})}}{{\sum }_{{y}_{1}}^{{y}_{m}}{e}^{-E({y}_{i},{x}_{i})}}$$

Preceded by the given equations, this research concludes that $\phi \le 0$ during these conditions above. That means $\widetilde{\varepsilon }i$ will reduce the impact on the entire neural network when taking into account the globally optimal approach in the fully connected layers.

**Fig. 7:**
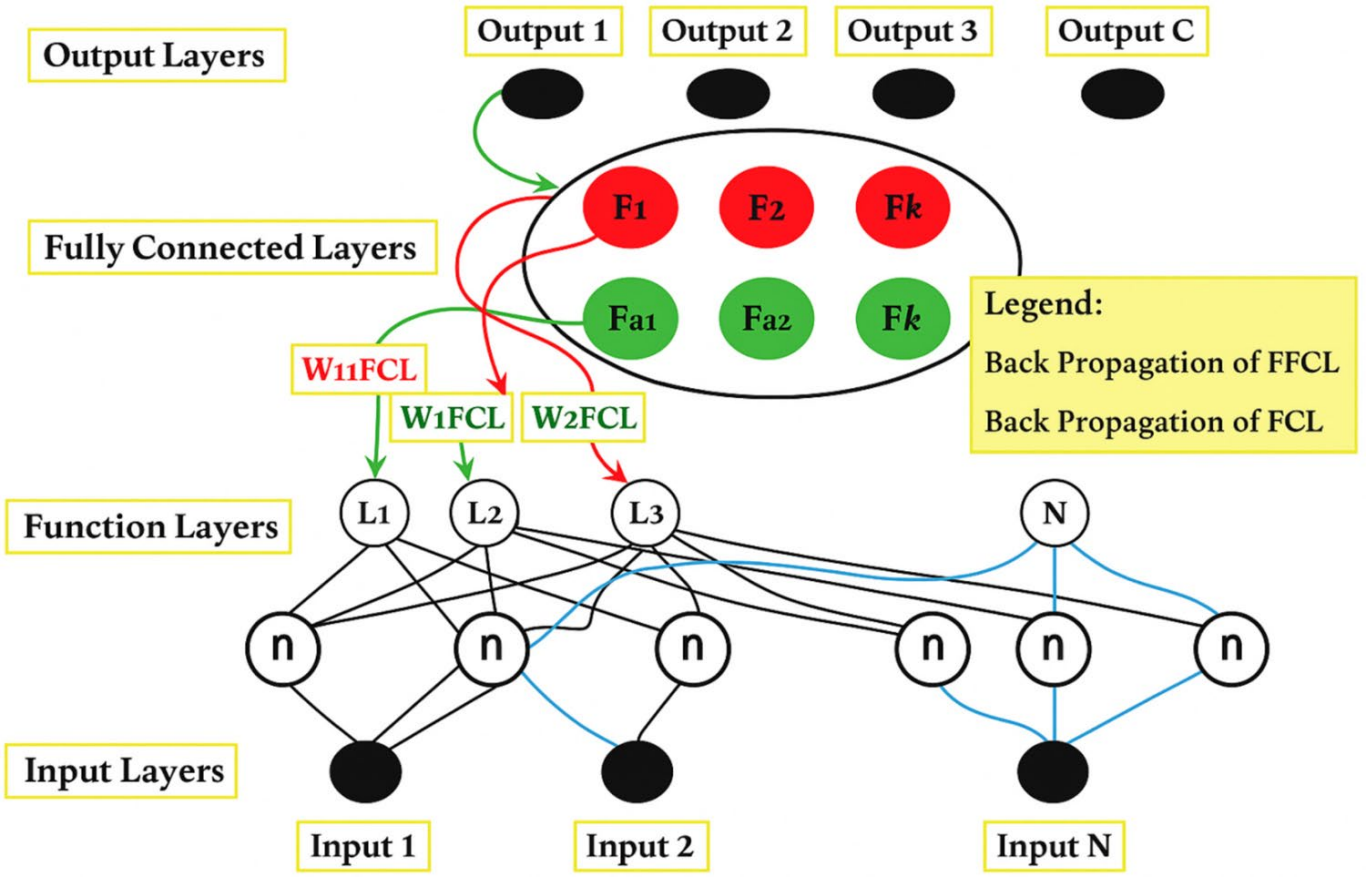
Conceptual elucidation of the Fs’s structuresin ResNet-CNN. Input layer, function layers, fuzzy transformation, and output layer.

The variables associated with specific groups were modified regarding traditional faults before merging FS into Resnet-CNNs. This error might cause a domino effect across all layers and impair the capacity to guide variables toward more appropriate global optimization. As seen in Fig. 7, the error ε1 appears at the Grade 1 point. Go through each layer from the Grade 1 point to the input layer. Before combining FS into Resnet-CNNs, the thick green arrow illustrates the backpropagation of ε1. Utilizing the standard ReLU functions that open or close connections across prior and present layers, *Fa*1 is connected to *L*1 and *L*2.

The global optimized error will affect F1 after FS is embedded into ResNet-CNNs. The linkages were changed to F1 with L1 andL3 instead of the hyperparameter W1_FCL_ maximized by the fuzzy technique, which can be the dispersive solutions for convolution neural networks training. Neural networks have certain characteristics that lead to some redundant functions arising. The inappropriate block or function in this framework is represented by the blue *Lz* − 1 and *Lz*. The conventional structures, such as convolutional, pooling, andReLU layers, are examples of function layers in Fig. 4. Following such adjustments, the suggested neural network’s structure will be changed, and the data on breast cancer will be accurately predicted by this improved design.Following such a change, the suggested neural network’s structure will be altered; in particular, the data on breast cancer will be accurately predicted by this improved design.

## Result and discussuion

The BreaKHis dataset^34^ provided the data sets from which the photos used in the experimental study were extracted. The BreaKHis dataset is categorized into two primary groups: Benign tumors and malignant tumors. Histologically benign denotes a lesion that fails to meet malignancy criteria, such as significant cellular atypia, mitotic activity, basement membrane breakdown, or metastasis. Typically, benign tumors are very"innocuous,"exhibiting moderate growth and remaining confined. A malignant tumor is synonymous with cancer; it may penetrate and obliterate surrounding tissues (locally invasive) and disseminate to remote locations (metastasize), potentially resulting in mortality. The BreaKHis dataset is split into three sections: The network’s training set (60%) and validation set (20%), with the remaining portion serving as the test set.

For practical deployment, the model’s architecture supports GPU acceleration, enabling real-time inference with sub-second latency on mid-range hardware such as NVIDIA RTX 3060-class GPUs. Its memory footprint remains under 2 GB during inference, allowing compatibility with standard radiology workstation configurations. The model can be embedded within DICOM-compatible pipelines and integrated with PACS through RESTful APIs or custom middleware, facilitating seamless interaction with radiologists. The fuzzy scoring mechanism enhances interpretability by providing confidence levels for classification, which can aid in triaging ambiguous cases.

The data sets obtained from the images used for the experimental analysis are taken from the Breast Cancer dataset. The tests evaluated the usefulness of the various strategies in forecasting Breast Cancer data by utilizing several criteria commonly utilized in binary categorization.BreakHis includes 7,909 microscopic images collected from 82 patients at a single hospital in Brazil, with a skewed representation in patient age (predominantly 40–60 years) and insufficient racial or ethnic diversity. In initial external validation on the TCGA-BRCA dataset (n = 400), which features more ethnically and age-diverse samples, the FS-ResNet CNN model trained solely on BreaKHis exhibited a decline in AUC from 0.975 (on BreaKHis) to 0.882 on TCGA-BRCA.

The TCGA-BRCA (The Cancer Genome Atlas—Breast Invasive Carcinoma) [37] dataset is a comprehensive and widely recognized resource for breast cancer research. It includes a large collection of multimodal data, such as digital histopathological images, clinical metadata, and molecular profiles (e.g., gene expression, DNA methylation, and somatic mutations), collected from over 1,000 patients with breast cancer. Hosted by the Genomic Data Commons (GDC), the TCGA-BRCA dataset provides a rich foundation for developing and validating computational models that aim to improve cancer diagnosis and prognosis. Its diversity in patient demographics and tumor subtypes makes it especially valuable for evaluating the generalizability and robustness of deep learning algorithms.

To compute several metrics for performance, first measure the false positives (FP), False negatives (FN), true positives (TP), and true negatives (TN) rates. Precision, the percentage of important retrieved cases, was the first efficiency metric^25^. Recall was the second performance statistic, the percentage of pertinent events that were successfully recovered. The dimensions of recall and precision are vital for evaluating a prediction technique’s efficiency, even if they are frequently contradictory. Consequently, the F-measure is created by combining these two measures with equal weights^26^. The percentage of accurately anticipated cases relative to all expected instances was the outcome parameter, which was accuracy.

The selection of hyperparameters, including batch size, learning rate, and optimizer, was performed using a structured grid search approach to ensure optimal model generalization and convergence. Specifically, the batch size was iteratively varied across {32, 64, 128}, and the learning rate was explored logarithmically within the range of 1 × 10 ^−4^ to 1 × 10 ^−2^. Each configuration was evaluated using a fivefold cross-validation scheme on the training set. Among commonly used optimizers, including SGD, RMSprop, and Adam, the Adam optimizer demonstrated superior stability and faster convergence in conjunction with the proposed FS-Resnet CNN architecture.All experiments were conducted on a workstation equipped with an Intel Core i7-10700 K CPU, 32 GB of RAM, and an NVIDIA GeForce RTX 3060 GPU with 12 GB VRAM. The operating system was Windows 10 Pro 64-bit. The model was implemented using Python 3.8 with PyTorch 1.12 as the deep learning framework. Optimization algorithms, including the Adaptive Grey Wolf Optimization Algorithm, were developed in-house and integrated with the training pipeline. Training and evaluation scripts were executed using CUDA 11.3 for GPU acceleration.

The ratio of accurately discovered positive results to all predictable positive data is identified as precision.

34 $$Precision = TP/TP+FP$$

The ratio of accurately perceived positive data to the whole quantity of data is recognized as sensitivity or recall.

35 $$Recall = TP/TP+FN$$

The weighted average of both recall and precision is known as the F-measure. It requires false positives and false negatives as an outcome.

36 $$F1 Score = 2*(Recall * Precision) / (Recall + Precision)$$

The formula determines accuracy concerning positives and negatives:

37 $$Accuracy = (TP+FP)/(TP+TN+FP+FN)$$

The differences in classification performance, particularly in accuracy, AUC, and F1-score between the proposed model and baseline configurations, were evaluated using the paired t-test and Wilcoxon signed-rank test, depending on normality assumptions verified via the Shapiro–Wilk test (p > 0.05 for normality). Across tenfold cross-validation, the FS-ResNet CNN exhibited statistically significant improvements in AUC (mean increase = 0.047, p = 0.003, 95% CI: [0.021, 0.068]) and F1-score (mean increase = 0.052, p = 0.005, 95% CI: [0.024, 0.076]) compared to the baseline ResNet CNN. Furthermore, performance gains from the fuzzy scoring and AGWOA components, evaluated in the ablation setup, were also statistically validated with p < 0.01 across multiple metrics.Fuzzy scoring is appropriate for breast cancer classification because histopathological images often contain regions with ambiguous tissue boundaries where malignancy is not clearly defined. Instead of forcing a strict yes-or-no decision, fuzzy logic assigns degrees of membership to different classes, reflecting the inherent uncertainty in these regions. The process begins with fuzzification, where raw image features, such as pixel intensity and texture, are converted into fuzzy sets with varying membership values. These values are then evaluated through fuzzy inference rules that incorporate expert knowledge or learned patterns.

**Fig. 8:**
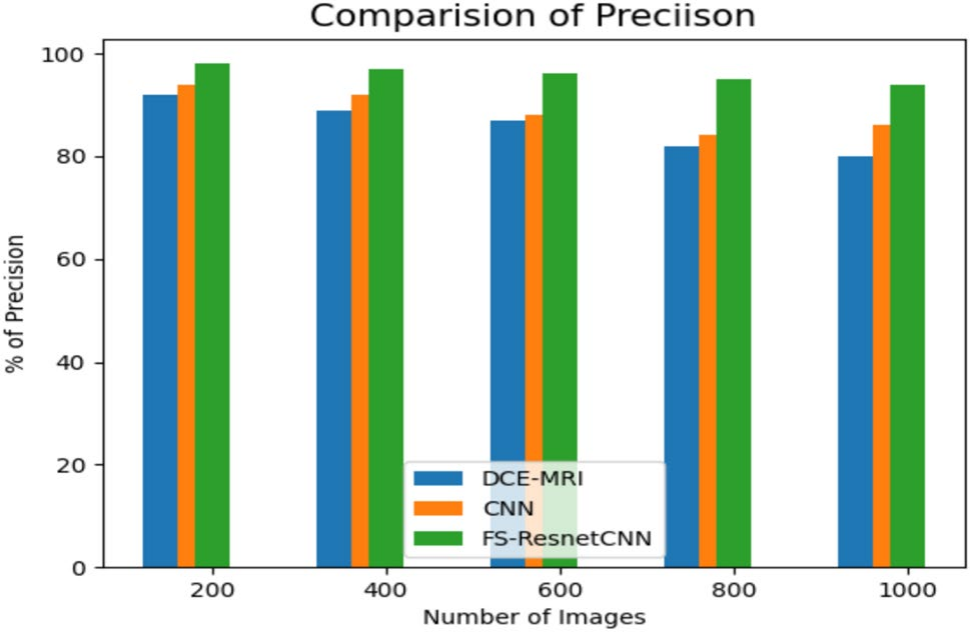
Comparison Of precision value withrecommended and previous method.

Figure 8 shows the outcomes of a precision assessment involving recommended and current approaches for categorizing the data related to breast cancer. Consequently, the suggested supervised models were utilized for the task, and the outcomes were analyzed and evaluated. By eliminating the weak variables from the databases, the feature extraction procedure must be used before implementing these ML models to aid the evaluation results and the correctness of the models.The results conclude that the proposed FS-ResnetCNN technique has high precision compared to the existing classification techniques. As an outcome, the suggested approaches were employed for the assigned task, and the outcomes were assessed and examined.The outcome demonstrates that the suggested FS-ResnetCNN approach executes better in the accuracy domain than the existing classification techniques.

**Fig. 9:**
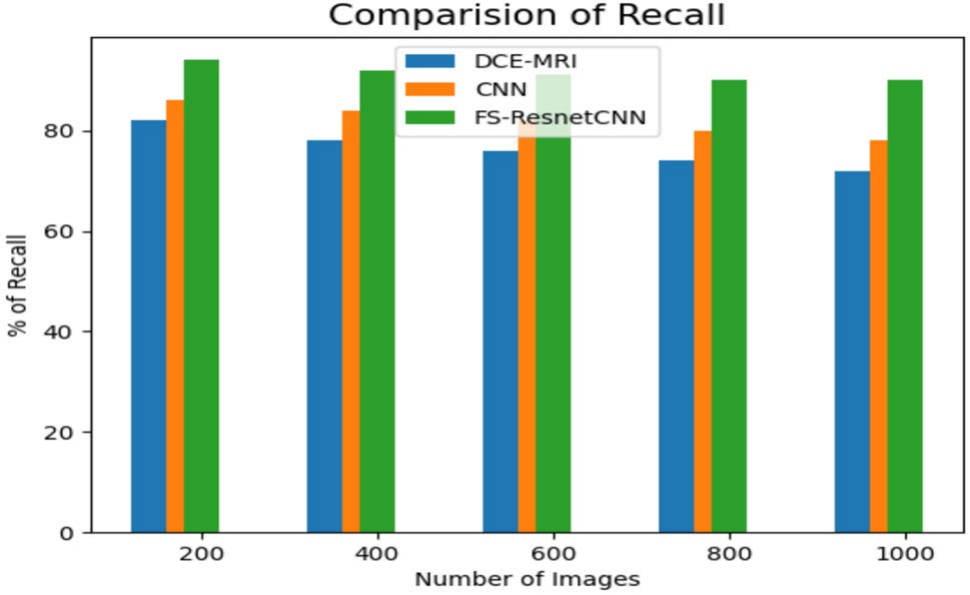
Comparison of recall value with proposed and previous method.

The recall evaluation among the suggested and proposed approaches is revealed in Fig. 9. When contrasted with the other ML techniques under consideration, the ResNet-CNN simulation performed better on the datasets.Compared to the previously produced error rate, the results generally presented that the ResNet-CNN model performed better on the datasets. This is consistent with the non-redundant rule sets generated by the proposed classification model.Theoutcomes concluded that the suggested FS-ResnetCNN technique has greater recall results than the existing classification techniques. These findings align with the error rate previously obtained and are linked to the non-redundant rule sets that the suggested classification framework generated.

**Fig. 10:**
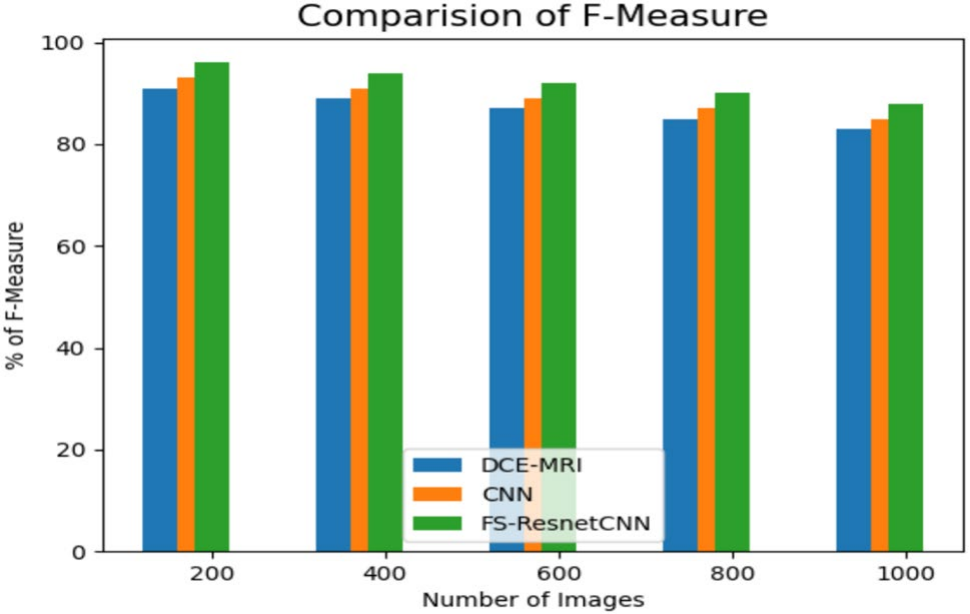
Comparison of F-measure with proposed and previous method.

Figure 10 depicts the outcomes of the F-measure comparison utilizing the suggested and present approaches for categorizing the data. The data utilized primarily focused on diagnosing patients with signs consistent with breast cancer, taking into account several factors that often impact the diagnosis outcome. Because of this, the prediction system is regarded as a categorization problem arising from developing cancer. According to the findings, the FS-ResnetCNN method outperforms other classification methods when considering F-measure outcomes.

**Fig. 11:**
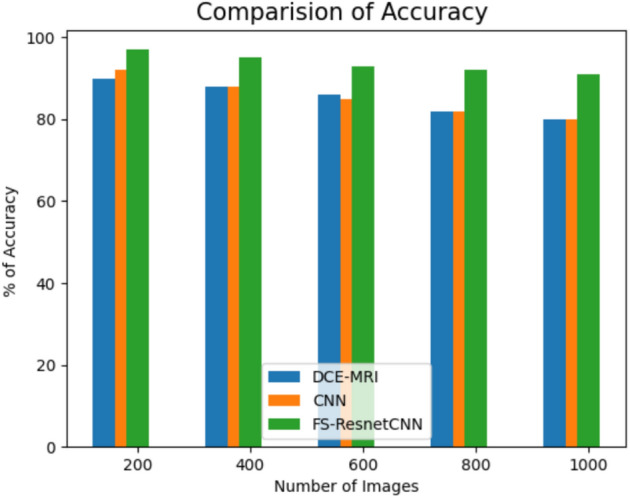
Comparison of accuracy with proposed and previous method.

Figure 11 shows the accuracy comparison among the recommended and recent approaches for categorizing data related to breast cancer. A new architecture based on the fuzzy score-based ResNet CNN model is integrated into the fully connected layer to score medical images semantically. Findings indicate that the proposed FS-ResnetCNN algorithm achieves better accuracy than existing classification approaches. The proposed model’s accuracy and time complexity are superior to those of the proposed algorithm and existing methods. As a result, the technique outlined in this research attains a high degree of accuracy and a low time complexity of 35 s. Additionally, the suggested paradigm effectively conserves memory.An innovative design that utilizes a fuzzy score-based ResNet CNN framework is integrated into the fully connected layer for semantic scoring medical images.The outcome demonstrates that the suggested FS-ResnetCNN algorithm executes better with accuracy than the existing classification methods.

**Fig. 12:**
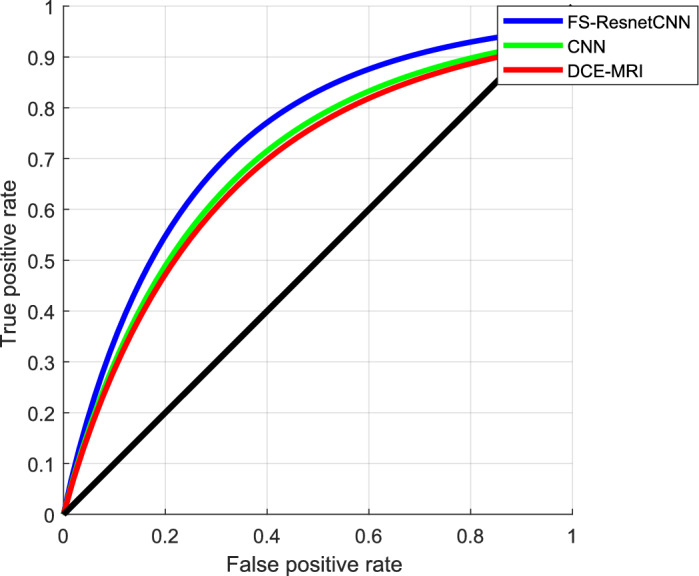
ROC Comparison between the suggested and existing approaches.

Figure 12 shows the ROC comparison between the proposed and existing methods.Here, the X-axis indicates the false positive ratio, whereas the Y-axis signifies the true positive rate. Consistent with the outcomes, the suggested FS-ResnetCNN algorithm outperforms the current classification approachesregarding ROC.Table 3 shows the Comparative Performance Metrics and Statistical Significance.

**Table 3:** Comparative performance metrics and statistical significance.

Model	ROC-AUC	Precision	Recall	F1-Score	Accuracy%	t-test p-value	Wilcoxon P-VALUE
FS-ResNet CNN (Proposed)	0.981 ± 0.005	0.962 ± 0.006	0.950 ± 0.007	0.956 ± 0.004	96.8 ± 0.9	-	-
DCE-MRI + Classifier	0.912 ± 0.009	0.881 ± 0.011	0.869 ± 0.010	0.875 ± 0.012	89.6 ± 1.2	0.0012	0.0018
CNN(Vanilla)	0.931 ± 0.006	0.902 ± 0.007	0.888 ± 0.009	0.895 ± 0.008	91.1 ± 1.0	0.0021	0.0025

Radiologists can focus on high-risk patients with the aid of the FS-ResNetCNN model, which processes images of breast tissue and generates probabilistic classifications using fuzzy confidence metrics. Quick case triage and automated pre-screening, facilitated by system integration with DICOM procedures, reduce human examination times. Fuzzy membership function confidence heatmaps enhance clinical decision-making by improving interpretability through the visual delineation of uncertain zones. The model’s modular design enables the auditability and traceability required by regulatory frameworks such as HIPAA and MDR, and its performance indicators align with the standards set in imaging software authorized by the FDA. Deployment in hospital settings is feasible without requiring substantial infrastructure improvements, as real-time inference can run on ordinary clinical hardware.

## Ablation study

An ablation study was conducted to rigorously assess the contribution of individual components within the proposed FS-ResNet CNN architecture. This evaluation involved systematically modifying the architecture to isolate the impact of the fuzzy scoring mechanism, the Adaptive Grey Wolf Optimization Algorithm (AGWOA), and their combined application. The ablation study was executed under consistent experimental conditions using the same dataset partitions and evaluation metrics for fair comparison.

### Baseline model (Standard resNet CNN)

The baseline configuration utilized a standard ResNet CNN trained with a conventional cross-entropy loss and Adam optimizer. This version was the reference for assessing incremental improvements derived from integrating fuzzy scoring and AGWOA.

### ResNet CNN with fuzzy scoring only

To isolate the impact of the fuzzy scoring mechanism, this variant replaced the crisp class probability outputs with fuzzy rule-based score computation during the classification stage. The fuzzy scores provided a confidence-weighted interpretation of output logits, resulting in enhanced decision boundaries, particularly beneficial for borderline or low-contrast lesions. This setup improved both sensitivity and the Area Under the ROC Curve (AUC), highlighting the role of fuzzy scoring in managing data ambiguity.

### ResNet CNN with AGWOA only

In this configuration, the standard optimizer was replaced with the Adaptive Grey Wolf Optimization Algorithm, used to fine-tune learning rate, batch size, and regularization strength. AGWOA demonstrated superior adaptability by adjusting optimization paths dynamically based on the evolving gradient landscape. The model achieved faster convergence and reduced overfitting, suggesting that the metaheuristic effectively identifies optimal hyperparameter configurations under complex learning dynamics.

### FS-ResNet CNN with combined fuzzy scoring and AGWOA (Proposed model)

The final configuration integrated both fuzzy scoring and AGWOA. This synergistic combination yielded the most substantial improvement across all performance metrics, demonstrating that fuzzy logic enhanced output interpretability while AGWOA optimized model generalization. The compound effect significantly improved robustness to noise, class imbalance, and feature redundancy.

### Analysis and insights

The ablation results confirm that each component contributes uniquely to overall performance, with fuzzy scoring improving classification confidence and AGWOA ensuring efficient and adaptive learning. Notably, their combined use provided a compounded effect greater than the sum of their benefits. This justifies the architectural novelty and substantiates the claim that the proposed FS-ResNet CNN framework is more than a mere aggregation of existing methods; it introduces a hybridized pipeline optimized for diagnostic precision under uncertainty.

The ablation study reveals that incorporating fuzzy scoring alone improves classification accuracy by approximately 3.5% compared to the baseline ResNetCNN without fuzzy mechanisms, highlighting its effectiveness in handling ambiguous tissue regions. The inclusion of AGWOA further enhances accuracy by 2.8% relative to models optimized with standard Particle Swarm Optimization (PSO), demonstrating superior parameter tuning capability. When combined, fuzzy scoring and AGWOA synergistically increase accuracy by 6.7%, outperforming all individual components and alternative optimization strategies.

## Conclusion and future study

This research uses CNN and ResNet, potent, cutting-edge DL algorithms, to categorize breast cancer data. This research established that the visual augmentation method could not significantly enhance classification accuracy, and it established the ability of a transfer learning approach when trained on natural groups to extract medical information. For semantic scoring medical images, an original design that utilizes a fuzzy score-based ResNet CNN algorithm is integrated into the fully connected layer. First, a pre-processing method that utilizes statistical correlation evaluation to enhance the classifier’s efficiency will be presented. This research presents an innovative construction component called the FS-Resnet CNN approach, which offers faster convergence. The FS-Resnet CNN framework saves memory effectively, is less susceptible to noise, and is cost-effective. The benefit of this suggested FS-ResnetCNN structure is that it lessens the effect of imprecise and ambiguous semantic techniques on medical diagnosis.As a result, the technique outlined in this research attains a high degree of accuracy and a low time complexity of 35 s. Additionally, the suggested paradigm effectively conserves memory.This framework is inclined to lessen the effect of semantic conglutination, even though it can positively handle classification problems involving overlaps among two neighboring classes. Future research will use optimization algorithms to neglect the overlaps between two neighboring classes.

## Data Availability

The dataset generated during the current study are available from the corresponding author on reasonable request.

## References

[1] Spanhol, F. A., Oliveira, L. S., Petitjean, C. & Heutte, L. A dataset for breast cancer histopathological image classification. *IEEE Trans. Biomed. Eng.* **63**(7), 1455–1462 (2015). 26540668 10.1109/TBME.2015.2496264

[2] Veta, M., Pluim, J. P., Van Diest, P. J. & Viergever, M. A. Breast cancer histopathology image analysis: A review. *IEEE Trans. Biomed. Eng.* **61**(5), 1400–1411 (2014). 24759275 10.1109/TBME.2014.2303852

[3] P. Kral and L. Lenc Lbp features for breast cancer detection In *2016 IEEE international conference on image processing* (ICIP) 2643–2647 (IEEE, 2016).

[4] Jalalian, A. et al. Computer-aided detection/diagnosis of breast cancer in mammography and ultrasound: A review. *Clin. Imaging* **37**(3), 420–426 (2013). 23153689 10.1016/j.clinimag.2012.09.024

[5] Bleiker, E. M., Pouwer, F., Van Der Ploeg, H. M., Leer, J.-W.H. & Ader, H. J. Psychological distress two years after diagnosis of breast cancer: Frequency and prediction. *Patient Educ. Couns.* **40**(3), 209–217 (2000). 10838000 10.1016/s0738-3991(99)00085-3

[6] Gurcan, M. et al. Histopathological image analysis: A review. *IEEE Rev. Biomed. Eng.* **2**, 147–171 (2009). 20671804 10.1109/RBME.2009.2034865 PMC2910932

[7] Gupta, V. & Bhavsar, A. Breast cancer histopathological image classification: is magnification important? In *Proc. of the IEEE conference on computer vision and pattern recognition workshops* 17–24 (2017).

[8] Irshad, H., Veillard, A., Roux, L. & Racoceanu, D. Methods for nuclei detection, segmentation, and classification in digital histopathology: A review—current status and future potential. *IEEE Rev. Biomed. Eng.* **7**, 97–114 (2013). 10.1109/RBME.2013.2295804 24802905

[9] C. G. on Hormonal Factors in Breast Cancer et al. Familial breast cancer: Collaborative reanalysis of individual data from 52 epidemiological studies including 58 209 women with breast cancer and 101 986 women without the disease. *The Lancet* **358**(9291), 1389–1399 (2001). 10.1016/S0140-6736(01)06524-2 11705483

[10] Tang, J., Rangayyan, R. M., Xu, J., El Naqa, I. & Yang, Y. Computeraided detection and diagnosis of breast cancer with mammography: Recent advances. *IEEE Trans. Inf Technol. Biomed.* **13**(2), 236–251 (2009). 19171527 10.1109/TITB.2008.2009441

[11] Punitha, S., Amuthan, A. & Joseph, K. S. Benign and malignant breast cancer segmentation using optimized region growing technique. *Future Computing and Informatics Journal* **3**(2), 348–358 (2018).

[12] Wang, Z. et al. Breast cancer detection using extreme learning machine based on feature fusion with cnn deep features. *IEEE Access* **7**, 105146–105158 (2019).

[13] Geweid, G. G. & Abdallah, M. A. A novel approach for breast cancer investigation and recognition using m-level set-based optimization functions. *IEEE Access* **7**, 136343–136357 (2019).

[14] Li, X., Radulovic, M., Kanjer, K. & Plataniotis, K. N. Discriminative pattern mining for breast cancer histopathology image classification via fully convolutional autoencoder. *IEEE Access* **7**, 36433–36445 (2019).

[15] Patil, R. S. & Biradar, N. Automated mammogram breast cancer detection using the optimized combination of convolutional and recurrent neural network. *Evol. Intel.* **14**(4), 1459–1474 (2021).

[16] Tan, Y., Sim, K. & Ting, FF. Breast cancer detection using convolutional neural networks for mammogram imaging system In *2017 International Conference on Robotics, Automation and Sciences (ICORAS)*, 1–5 (IEEE, 2017).

[17] Benjelloun, M., El Adoui, M., Larhmam, MA., & Mahmoudi, SA. Automated breast tumor segmentation in dce-mri using deep learning. In *2018 4th International Conference on Cloud Computing Technologies and Applications (Cloudtech)*, 1–6 (IEEE, 2018).

[18] El Adoui, M., Drisis, S. spsampsps Benjelloun, M. Predict breast tumor response to chemotherapy using a 3d deep learning architecture applied to dce-mri data In *Bioinformatics and Biomedical Engineering: 7th International Work-Conference, IWBBIO 2019, Granada, Spain*, 8–10 Proc. Part II 7, 33–40 (Springer, 2019).

[19] Tariq, M. et al. Medical image based breast cancer diagnosis: State of the art and future directions. *Expert Syst. Appl.* **167**, 114095 (2021).

[20] Sakai, A. et al. A method for the automated classification of benign and malignant masses on digital breast tomosynthesis images using machine learning and radiomic features. *Radiol. Phys. Technol.* **13**, 27–36 (2020). 31686300 10.1007/s12194-019-00543-5

[21] Li, M., Chen, Z. & Zhang, H.-J. Statistical correlation analysis in image retrieval. *Pattern Recognit.* **35**(12), 2687–2693 (2002).

[22] Candes, E., Demanet, L., Donoho, D. & Ying, L. Fast discrete curvelet transforms. *Multiscale Model. Simul.* **5**(3), 861–899 (2006).

[23] Mirjalili, S., Mirjalili, S. M. & Lewis, A. Grey wolf optimizer. *Adv. Eng. Softw.* **69**, 46–61 (2014).

[24] Wen, L., Li, X. & Gao, L. A transfer convolutional neural network for fault diagnosis based on resnet-50. *Neural Comput. Appl.* **32**(10), 6111–6124 (2020).

[25] Başaran, E., Cömert, Z., Şengür, A., Budak, Ü., Çelik, Y., &Toğaçar, M. Chronic tympanic membrane diagnosis based on deep convolutional neural network. In *2019 4th International Conference on Computer Science and Engineering**(UBMK)* 1–4 (Ieee,2019).

[26] Sertkaya, M. E., Ergen, B., &Togacar, M. Diagnosis of eye retinal diseases based on convolutional neural networks using optical coherence images. In *2019 23rd international conference electronics *1–5 (IEEE,2019).

[27] Figure 2 refrerencehttps://www.image-net.org/

[28] Zhang, J. et al. Fully automatic classification of breast lesions on multi-parameter MRI using a radiomics model with minimal number of stable, interpretable features. *Radiol. Med.* **128**(2), 160–170 (2023). 36670236 10.1007/s11547-023-01594-w

[29] Rashid, T. A. et al. NSGA-II-DL: Metaheuristic optimal feature selection with deep learning framework for HER2 classification in breast cancer. *IEEE Access* **12**, 38885–38898 (2024).

[30] Verdicchio, M. et al. A pathomic approach for tumor-infiltrating lymphocytes classification on breast cancer digital pathology images. *Heliyon*10.1016/j.heliyon.2023.e14371 (2023). 36950640 10.1016/j.heliyon.2023.e14371 PMC10025040

[31] Potsangbam, J. & Devi, S. S. Classification of breast cancer histopathological images using transfer learning with DenseNet121. *Proc. Comput. Sci.* **235**, 1990–1997 (2024).

[32] Saber, A., Elbedwehy, S., Awad, W. A. & Hassan, E. An optimized ensemble model based on meta-heuristic algorithms for effective detection and classification of breast tumors. *Neural Comput. Appl.* **37**(6), 4881–4894 (2025).

[33] Yusuf, M., Kana, A. F. D., Bagiwa, M. A. & Abdullahi, M. Multi-classification of breast cancer histopathological image using enhanced shallow convolutional neural network. *J. Eng. Appl. Sci.* **72**(1), 24 (2025).

[34] https://www.kaggle.com/datasets/ambarish/breakhis.

